# Supplementary material for: Steroids from the Deep-Sea-Derived Fungus Penicillium granulatum MCCC 3A00475 Induced Apoptosis via Retinoid X Receptor (RXR)-α Pathway
Source: Mar Drugs. 2019 Mar 19;17(3):178. doi: 10.3390/md17030178 (PMC6472029; doi:10.3390/md17030178)

Supplementary Materials

Steroids from the Deep-Sea-Derived Fungus *Penicillium granulatum* MCCC 3A00475 Induced Apoptosis via Retinoid X Receptor (RXR)-*α* Pathway

Chun-Lan Xie [1,2,†], Duo Zhang [1,†], Jin-Mei Xia [2], Chao-Chao Hu [1], Ting Lin [1], Yu-Kun Lin [2], Guang-Hui Wang [1] Wen-Jing Tian [1], Zeng-Peng Li [2], Xiao-Kun Zhang [1,*], Xian-Wen Yang [2,*] and Hai-Feng Chen [1,*]

[1] School of Pharmaceutical Sciences, Xiamen University, South Xiangan Road, Xiamen, 361005, China; xiechunlanxx@163.com (C.-L.X.); 32320151154210@stu.xmu.edu.cn (D.Z.); 32320171153261@stu.xmu.edu.cn (C.-C.H.); linting@xmu.edu.cn (T.L.); guanghui@xmu.edu.cn (G.-H.W.); tianwj@xmu.edu.cn (W.-J.T.)

[2] Key Laboratory of Marine Biogenetic Resources, South China Sea Bio-Resource Exploitation and Utilization Collaborative Innovation Center, Third Institute of Oceanography, Ministry of Natural Resources, 184 Daxue Road, Xiamen 361005, China; xiajinmei@tio.org.cn (J.-M.X.); yukunlin223@163.com (Y.-K.L.); lizengpeng@tio.org.cn (Z.-P.L.)

* Correspondence: xkzhang@xmu.edu.cn (X.-K.Z.); yangxianwen@tio.org.cn (X.-W.Y.); haifeng@xmu.edu.cn (H.-F.C.); Tel.: +86-592-2181851 (X.-K.Z.); +86-592-2195319 (X.-W.Y.); +86-592-592-2187225 (H.-F.C.)

† These authors contributed equally to this work.

Contents

Figure S1. ^{1}H-NMR spectrum of penicisteroid D (**1**);

Figure S2. ^{13}C-NMR spectrum of penicisteroid D (**1**);

Figure S3. HSQC spectrum of the penicisteroid D (**1**);

Figure S4. COSY spectrum of penicisteroid D (**1**);

Figure S5. HMBC spectrum of penicisteroid D (**1**);

Figure S6. NOESY spectrum of penicisteroid D (**1**).

Figure S7. ^{1}H-NMR spectrum of penicisteroid E (**2**);

Figure S8. ^{13}C-NMR spectrum of penicisteroid E (**2**);

Figure S9. HSQC spectrum of penicisteroid E (**2**);

Figure S10. COSY spectrum of penicisteroid E (**2**);

Figure S11. HMBC spectrum of penicisteroid E (**2**);

Figure S12. NOESY spectrum of penicisteroid E (**2**).

Figure S13. ^{1}H-NMR spectrum of penicisteroid F (**3**);

Figure S14. ^{13}C-NMR spectrum of penicisteroid F (**3**);

Figure S15. HSQC spectrum of penicisteroid F (**3**);

Figure S16. COSY spectrum of penicisteroid F (**3**);

Figure S17. HMBC spectrum of penicisteroid F (**3**);

Figure S18. NOESY spectrum of penicisteroid F (**3**).

Figure S19. ^{1}H-NMR spectrum of penicisteroid G (**4**);

Figure S20. ^{13}C-NMR spectrum of penicisteroid G (**4**);

Figure S21. HSQC spectrum of penicisteroid G (**4**);

Figure S22. COSY spectrum of penicisteroid G (**4**);

Figure S23. HMBC spectrum of penicisteroid G (**4**);

Figure S24. NOESY spectrum of penicisteroid G (**4**).

Figure S25. ^{1}H-NMR spectrum of penicisteroid H (**5**);

Figure S26. ^{13}C-NMR spectrum of penicisteroid H (**5**);

Figure S27. HSQC spectrum of penicisteroid H (**5**);

Figure S28. COSY spectrum of penicisteroid H (**5**);

Figure S29. HMBC spectrum of penicisteroid H (**5**);

Figure S30. NOESY spectrum of penicisteroid H (**5**).

Table S1. Crystal data and structure refinement for penicisteroid D (**1**).

Identification code	penicisteroid D	
Empirical formula	C30 H48 O3	
Formula weight	456.68	
Temperature	99.98(11) K	
Wavelength	1.54184 Å	
Crystal system	Orthorhombic	
Space group	$P2_12_12_1$	
Unit cell dimensions	a = 5.94350(10) Å	α = 90°.
	b = 11.98440(10) Å	β = 90°
	c = 38.3459(4) Å	γ = 90°.
Volume	2731.35(6) $Å^3$	
Z	4	
Density (calculated)	1.111 Mg/m3	
Absorption coefficient	0.533 mm−1	
F(000)	1008	
Crystal size	0.15 × 0.12 × 0.05 mm3	
Theta range for data collection	2.304 to 67.080°	
Index ranges	−6<=h<=7, −14<=k<=14, −45<=l<=45	
Reflections collected	18704	
Independent reflections	4726 [R(int) = 0.0845]	
Completeness to theta = 67.080°	98.10%	
Absorption correction	Semi-empirical from equivalents	
Max. and min. transmission	1.00000 and 0.56522	
Refinement method	Full-matrix least-squares on F2	
Data / restraints / parameters	4726/0/306	
Goodness-of-fit on F2	1.091	
Final R indices [I>2sigma(I)]	R1 = 0.0797, wR2 = 0.2359	
R indices (all data)	R1 = 0.0842, wR2 = 0.2490	
Absolute structure parameter	−0.06(15)	
Extinction coefficient	n/a	
Largest diff. peak and hole	0.484 and -0.353 e.$Å^{-3}$	

Table S2. Atomic coordinates (×10⁴) and equivalent isotropic displacement parameters (Å^2 × 10^3) for penicisteroid D. U(eq) is defined as one third of the trace of the orthogonalized U^{ij} tensor.

	**x**	**y**	**z**	**U(eq)**
O(001)	−1724(6)	−3598(2)	−6072(1)	33(1)
O(002)	−3214(7)	−5300(3)	−8968(1)	39(1)
O(003)	−4416(8)	−2368(3)	−5934(1)	46(1)
C(004)	−2069(7)	−4346(3)	−7288(1)	28(1)
C(005)	−2605(8)	−4365(3)	−8043(1)	31(1)
C(006)	−3790(8)	−3575(3)	−7462(1)	32(1)
C(007)	−2782(7)	−4605(3)	−6913(1)	30(1)
C(008)	−3289(8)	−3629(3)	−6666(1)	31(1)
C(009)	−1311(9)	−5884(4)	−6023(1)	33(1)
C(00A)	−3268(8)	−4184(3)	−6304(1)	32(1)
C(00B)	−1165(8)	−5362(3)	−6707(1)	30(1)
C(00C)	−3292(8)	−5425(4)	−8600(1)	34(1)
C(00D)	−3693(8)	−3605(3)	−7852(1)	32(1)
C(00E)	−2658(9)	−4313(4)	−8435(1)	35(1)
C(00F)	−1913(7)	−5463(3)	−7488(1)	30(1)
C(00G)	−3305(9)	−5345(4)	−5461(1)	34(1)
C(00H)	−2523(10)	−2716(4)	−5900(1)	39(1)
C(00I)	−1038(9)	−6470(4)	−6907(1)	32(1)
C(00J)	−1277(8)	−5319(4)	−7876(1)	32(1)
C(00K)	1191(8)	−4849(4)	−6664(1)	33(1)
C(00L)	−260(10)	−7029(4)	−6080(1)	42(1)
C(00M)	−1730(9)	−6334(4)	−8470(1)	36(1)
C(00N)	−781(11)	−2226(4)	−5661(1)	44(1)
C(00O)	−5211(9)	−5404(4)	−5204(1)	36(1)
C(00P)	−1849(9)	−6414(4)	−8073(1)	32(1)
C(00Q)	−4386(9)	−5467(4)	−4825(1)	35(1)
C(00R)	−2432(8)	−5396(4)	−6354(1)	31(1)
C(00S)	−2619(10)	−6381(4)	−4777(1)	43(1)
C(00T)	−3120(10)	−5966(4)	−5745(1)	36(1)
C(00U)	−371(8)	−6287(4)	−7291(1)	34(1)
C(00V)	1247(9)	−5056(4)	−7923(1)	36(1)
C(00W)	−6318(10)	−5667(4)	−4569(1)	40(1)
C(00X)	−6773(10)	−4399(5)	−5265(1)	45(1)

Table S3. Bond lengths [Å] and angles [°] for penicisteroid D.

O(001)-C(00A)	1.459(6)	C(00H)-O(001)-C(00A)	117.4(4)	C(00V)-C(00J)-C(00F)	111.9(4)
O(001)-C(00H)	1.332(6)	C(00C)-O(002)-H(002)	109.5	C(00V)-C(00J)-C(00P)	109.2(4)
O(002)-H(002)	0.84	C(006)-C(004)-H(004)	109.5	C(00B)-C(00K)-H(00N)	109.5
O(002)-C(00C)	1.421(5)	C(006)-C(004)-C(007)	110.3(4)	C(00B)-C(00K)-H(00O)	109.5
O(003)-C(00H)	1.207(7)	C(006)-C(004)-C(00F)	110.3(4)	C(00B)-C(00K)-H(00P)	109.5
C(004)-H(004)	1	C(007)-C(004)-H(004)	109.5	H(00N)-C(00K)-H(00O)	109.5
C(004)-C(006)	1.530(6)	C(007)-C(004)-C(00F)	107.8(3)	H(00N)-C(00K)-H(00P)	109.5
C(004)-C(007)	1.532(6)	C(00F)-C(004)-H(004)	109.5	H(00O)-C(00K)-H(00P)	109.5
C(004)-C(00F)	1.545(6)	C(00D)-C(005)-C(00E)	120.6(4)	C(009)-C(00L)-H(00Q)	109.5
C(005)-C(00D)	1.335(7)	C(00D)-C(005)-C(00J)	122.0(4)	C(009)-C(00L)-H(00R)	109.5
C(005)-C(00E)	1.503(7)	C(00E)-C(005)-C(00J)	117.4(4)	C(009)-C(00L)-H(00S)	109.5
C(005)-C(00J)	1.529(6)	C(004)-C(006)-H(00A)	108.9	H(00Q)-C(00L)-H(00R)	109.5
C(006)-H(00A)	0.99	C(004)-C(006)-H(00B)	108.9	H(00Q)-C(00L)-H(00S)	109.5
C(006)-H(00B)	0.99	H(00A)-C(006)-H(00B)	107.7	H(00R)-C(00L)-H(00S)	109.5
C(006)-C(00D)	1.499(6)	C(00D)-C(006)-C(004)	113.2(4)	C(00C)-C(00M)-H(00T)	109.6
C(007)-H(007)	1	C(00D)-C(006)-H(00A)	108.9	C(00C)-C(00M)-H(00U)	109.6
C(007)-C(008)	1.535(6)	C(00D)-C(006)-H(00B)	108.9	C(00C)-C(00M)-C(00P)	110.1(4)
C(007)-C(00B)	1.539(6)	C(004)-C(007)-H(007)	105.6	H(00T)-C(00M)-H(00U)	108.1
C(008)-H(00C)	0.99	C(004)-C(007)-C(008)	118.7(3)	C(00P)-C(00M)-H(00T)	109.6
C(008)-H(00D)	0.99	C(004)-C(007)-C(00B)	115.4(4)	C(00P)-C(00M)-H(00U)	109.6
C(008)-C(00A)	1.540(6)	C(008)-C(007)-H(007)	105.6	C(00H)-C(00N)-H(00V)	109.5
C(009)-H(009)	1	C(008)-C(007)-C(00B)	104.8(3)	C(00H)-C(00N)-H(00W)	109.5
C(009)-C(00L)	1.525(7)	C(00B)-C(007)-H(007)	105.6	C(00H)-C(00N)-H(00X)	109.5
C(009)-C(00R)	1.548(6)	C(007)-C(008)-H(00C)	111.2	H(00V)-C(00N)-H(00W)	109.5
C(009)-C(00T)	1.515(7)	C(007)-C(008)-H(00D)	111.2	H(00V)-C(00N)-H(00X)	109.5
C(00A)-H(00E)	1	C(007)-C(008)-C(00A)	103.1(3)	H(00W)-C(00N)-H(00X)	109.5
C(00A)-C(00R)	1.547(6)	H(00C)-C(008)-H(00D)	109.1	C(00G)-C(00O)-H(00Y)	107.9
C(00B)-C(00I)	1.534(6)	C(00A)-C(008)-H(00C)	111.2	C(00G)-C(00O)-C(00Q)	112.5(4)
C(00B)-C(00K)	1.538(6)	C(00A)-C(008)-H(00D)	111.2	C(00G)-C(00O)-C(00X)	108.5(4)
C(00B)-C(00R)	1.552(6)	C(00L)-C(009)-H(009)	108.9	C(00Q)-C(00O)-H(00Y)	107.9
C(00C)-H(00F)	1	C(00L)-C(009)-C(00R)	113.4(4)	C(00Q)-C(00O)-C(00X)	112.1(4)
C(00C)-C(00E)	1.522(6)	C(00R)-C(009)-H(009)	108.9	C(00X)-C(00O)-H(00Y)	107.9
C(00C)-C(00M)	1.515(7)	C(00T)-C(009)-H(009)	108.9	C(00J)-C(00P)-H(00Z)	108.6
C(00D)-H(00G)	0.95	C(00T)-C(009)-C(00L)	109.5(4)	C(00J)-C(00P)-H	108.6
C(00E)-H(00H)	0.99	C(00T)-C(009)-C(00R)	107.1(4)	C(00M)-C(00P)-C(00J)	114.8(4)
C(00E)-H(00I)	0.99	O(001)-C(00A)-C(008)	110.4(4)	C(00M)-C(00P)-H(00Z)	108.6
C(00F)-H(00J)	1	O(001)-C(00A)-H(00E)	110.1	C(00M)-C(00P)-H	108.6

C(00F)-C(00J)	1.547(6)	O(001)-C(00A)-C(00R)	109.0(4)	H(00Z)-C(00P)-H	107.5
C(00F)-C(00U)	1.545(6)	C(008)-C(00A)-H(00E)	110.1	C(00O)-C(00Q)-H(00)	108
C(00G)-H(00K)	0.95	C(008)-C(00A)-C(00R)	107.3(3)	C(00S)-C(00Q)-C(00O)	111.6(4)
C(00G)-C(00O)	1.504(7)	C(00R)-C(00A)-H(00E)	110.1	C(00S)-C(00Q)-H(00)	108
C(00G)-C(00T)	1.324(7)	C(007)-C(00B)-C(00R)	99.2(3)	C(00S)-C(00Q)-C(00W)	109.1(4)
C(00H)-C(00N)	1.501(8)	C(00I)-C(00B)-C(007)	106.6(3)	C(00W)-C(00Q)-C(00O)	112.0(4)
C(00I)-H(00L)	0.99	C(00I)-C(00B)-C(00K)	110.8(4)	C(00W)-C(00Q)-H(00)	108
C(00I)-H(00M)	0.99	C(00I)-C(00B)-C(00R)	115.9(3)	C(009)-C(00R)-C(00B)	121.2(4)
C(00I)-C(00U)	1.541(6)	C(00K)-C(00B)-C(007)	112.8(3)	C(009)-C(00R)-H(0AA)	105.9
C(00J)-C(00P)	1.551(6)	C(00K)-C(00B)-C(00R)	111.0(4)	C(00A)-C(00R)-C(009)	113.1(4)
C(00J)-C(00V)	1.543(7)	O(002)-C(00C)-H(00F)	108.7	C(00A)-C(00R)-C(00B)	103.8(3)
C(00K)-H(00N)	0.98	O(002)-C(00C)-C(00E)	108.2(3)	C(00A)-C(00R)-H(0AA)	105.9
C(00K)-H(00O)	0.98	O(002)-C(00C)-C(00M)	112.4(4)	C(00B)-C(00R)-H(0AA)	105.9
C(00K)-H(00P)	0.98	C(00E)-C(00C)-H(00F)	108.7	C(00Q)-C(00S)-H(1AA)	109.5
C(00L)-H(00Q)	0.98	C(00M)-C(00C)-H(00F)	108.7	C(00Q)-C(00S)-HA	109.5
C(00L)-H(00R)	0.98	C(00M)-C(00C)-C(00E)	110.0(4)	C(00Q)-C(00S)-HB	109.5
C(00L)-H(00S)	0.98	C(005)-C(00D)-C(006)	125.7(4)	H(1AA)-C(00S)-HA	109.5
C(00M)-H(00T)	0.99	C(005)-C(00D)-H(00G)	117.2	H(1AA)-C(00S)-HB	109.5
C(00M)-H(00U)	0.99	C(006)-C(00D)-H(00G)	117.2	HA-C(00S)-HB	109.5
C(00M)-C(00P)	1.528(6)	C(005)-C(00E)-C(00C)	112.5(3)	C(009)-C(00T)-H(2AA)	116.6
C(00N)-H(00V)	0.98	C(005)-C(00E)-H(00H)	109.1	C(00G)-C(00T)-C(009)	126.8(5)
C(00N)-H(00W)	0.98	C(005)-C(00E)-H(00I)	109.1	C(00G)-C(00T)-H(2AA)	116.6
C(00N)-H(00X)	0.98	C(00C)-C(00E)-H(00H)	109.1	C(00F)-C(00U)-H(3AA)	108.8
C(00O)-H(00Y)	1	C(00C)-C(00E)-H(00I)	109.1	C(00F)-C(00U)-HC	108.8
C(00O)-C(00Q)	1.537(7)	H(00H)-C(00E)-H(00I)	107.8	C(00I)-C(00U)-C(00F)	114.0(4)
C(00O)-C(00X)	1.539(7)	C(004)-C(00F)-H(00J)	106.4	C(00I)-C(00U)-H(3AA)	108.8
C(00P)-H(00Z)	0.99	C(004)-C(00F)-C(00J)	113.3(3)	C(00I)-C(00U)-HC	108.8
C(00P)-H	0.99	C(00J)-C(00F)-H(00J)	106.4	H(3AA)-C(00U)-HC	107.7
C(00Q)-H(00)	1	C(00U)-C(00F)-C(004)	110.3(4)	C(00J)-C(00V)-H(4AA)	109.5
C(00Q)-C(00S)	1.528(7)	C(00U)-C(00F)-H(00J)	106.4	C(00J)-C(00V)-HD	109.5
C(00Q)-C(00W)	1.530(7)	C(00U)-C(00F)-C(00J)	113.4(4)	C(00J)-C(00V)-HE	109.5
C(00R)-H(0AA)	1	C(00O)-C(00G)-H(00K)	117.4	H(4AA)-C(00V)-HD	109.5
C(00S)-H(1AA)	0.98	C(00T)-C(00G)-H(00K)	117.4	H(4AA)-C(00V)-HE	109.5
C(00S)-HA	0.98	C(00T)-C(00G)-C(00O)	125.2(5)	HD-C(00V)-HE	109.5
C(00S)-HB	0.98	O(001)-C(00H)-C(00N)	111.4(5)	C(00Q)-C(00W)-H(5AA)	109.5
C(00T)-H(2AA)	0.95	O(003)-C(00H)-O(001)	123.6(5)	C(00Q)-C(00W)-HF	109.5
C(00U)-H(3AA)	0.99	O(003)-C(00H)-C(00N)	125.0(5)	C(00Q)-C(00W)-HG	109.5
C(00U)-HC	0.99	C(00B)-C(00I)-H(00L)	109.3	H(5AA)-C(00W)-HF	109.5

C(00V)-H(4AA)	0.98	C(00B)-C(00I)-H(00M)	109.3	H(5AA)-C(00W)-HG	109.5
C(00V)-HD	0.98	C(00B)-C(00I)-C(00U)	111.4(3)	HF-C(00W)-HG	109.5
C(00V)-HE	0.98	H(00L)-C(00I)-H(00M)	108	C(00O)-C(00X)-H(6AA)	109.5
C(00W)-H(5AA)	0.98	C(00U)-C(00I)-H(00L)	109.3	C(00O)-C(00X)-HH	109.5
C(00W)-HF	0.98	C(00U)-C(00I)-H(00M)	109.3	C(00O)-C(00X)-HI	109.5
C(00W)-HG	0.98	C(005)-C(00J)-C(00F)	111.1(4)	H(6AA)-C(00X)-HH	109.5
C(00X)-H(6AA)	0.98	C(005)-C(00J)-C(00P)	108.4(4)	H(6AA)-C(00X)-HI	109.5
C(00X)-HH	0.98	C(005)-C(00J)-C(00V)	107.5(4)	HH-C(00X)-HI	109.5
C(00X)-HI	0.98	C(00F)-C(00J)-C(00P)	108.6(3)		

Symmetry transformations used to generate equivalent atoms.

Table S4. Anisotropic displacement parameters (Å^2 × 10^3) for penicisteroid D. The anisotropic displacement factor exponent takes the form: $-2\pi^2$[h^2 a*^{2}U^{11} + ... + 2 h k a* b* U^{12}].

	U11	U22	U33	U23	U13	U12
O(001)	35(2)	31(1)	33(2)	−3(1)	−2(1)	0(1)
O(002)	46(2)	38(2)	33(2)	1(1)	3(1)	−6(2)
O(003)	54(2)	40(2)	45(2)	−8(1)	−4(2)	14(2)
C(004)	24(2)	28(2)	32(2)	1(2)	−1(2)	0(2)
C(005)	26(2)	28(2)	39(2)	1(2)	2(2)	−5(2)
C(006)	28(2)	27(2)	40(2)	−3(2)	−2(2)	3(2)
C(007)	23(2)	27(2)	40(2)	−3(2)	2(2)	−2(2)
C(008)	30(2)	27(2)	36(2)	−1(2)	−2(2)	2(2)
C(009)	38(3)	34(2)	28(2)	0(2)	0(2)	0(2)
C(00A)	31(2)	32(2)	32(2)	−3(2)	1(2)	−1(2)
C(00B)	32(2)	29(2)	28(2)	0(2)	4(2)	0(2)
C(00C)	32(2)	35(2)	35(2)	1(2)	1(2)	−5(2)
C(00D)	33(2)	28(2)	34(2)	3(2)	−2(2)	−2(2)
C(00E)	36(2)	30(2)	39(2)	4(2)	1(2)	−1(2)
C(00F)	22(2)	29(2)	40(2)	1(2)	0(2)	−1(2)
C(00G)	38(2)	32(2)	32(2)	2(2)	1(2)	1(2)
C(00H)	55(3)	27(2)	34(2)	0(2)	−5(2)	3(2)
C(00I)	34(2)	29(2)	35(2)	0(2)	−1(2)	3(2)
C(00J)	31(2)	30(2)	36(2)	−1(2)	1(2)	2(2)
C(00K)	28(2)	35(2)	36(2)	1(2)	−1(2)	3(2)
C(00L)	50(3)	38(2)	38(2)	2(2)	0(2)	8(2)
C(00M)	40(3)	31(2)	36(2)	−3(2)	5(2)	−2(2)
C(00N)	57(3)	34(2)	41(3)	−5(2)	−6(2)	−1(2)
C(00O)	38(2)	34(2)	37(2)	−2(2)	2(2)	−1(2)
C(00P)	38(2)	30(2)	29(2)	0(2)	2(2)	2(2)
C(00Q)	41(3)	31(2)	32(2)	−1(2)	−3(2)	−1(2)
C(00R)	27(2)	30(2)	35(2)	−2(2)	1(2)	−3(2)
C(00S)	46(3)	43(2)	40(2)	4(2)	0(2)	6(2)
C(00T)	45(3)	33(2)	30(2)	2(2)	0(2)	−1(2)
C(00U)	37(3)	30(2)	35(2)	−2(2)	4(2)	7(2)
C(00V)	33(3)	46(2)	29(2)	−1(2)	−2(2)	0(2)
C(00W)	48(3)	38(2)	35(2)	0(2)	5(2)	−3(2)
C(00X)	41(3)	52(3)	41(3)	4(2)	2(2)	9(2)

Table S5. Hydrogen coordinates (×10^4) and isotropic displacement parameters (Å^2 × 10^3) for penicisteroid D.

	x	y	z	U(eq)		x	y	z	U(eq)
H(002)	−3899	−5833	−9062	58	H(00U)	−2168	−7057	−8574	43
H(004)	−562	−3975	−7287	34	H(00V)	542	−2714	−5656	66
H(00A)	−5319	−3794	−7386	38	H(00W)	−1405	−2158	−5426	66
H(00B)	−3523	−2801	−7382	38	H(00X)	−344	−1487	−5747	66
H(007)	−4220	−5033	−6933	36	H(00Y)	−6089	−6097	−5254	44
H(00C)	−4776	−3296	−6717	38	H(00Z)	−797	−7003	−7994	39
H(00D)	−2120	−3044	−6683	38	H	−3387	−6649	−8006	39
H(009)	−118	−5356	−5941	40	H(00)	−3672	−4736	−4765	42
H(00E)	−4819	−4185	−6203	38	H(0AA)	−3810	−5856	−6395	37
H(00F)	−4867	−5616	−8530	41	H(1AA)	−3277	−7106	−4837	64
H(00G)	−4472	−3034	−7975	38	HA	−2115	−6392	−4534	64
H(00H)	−1158	−4084	−8521	42	HB	−1333	−6231	−4930	64
H(00I)	−3757	−3739	−8509	42	H(2AA)	−4238	−6522	−5779	43
H(00J)	−3456	−5795	−7484	36	H(3AA)	−400	−7014	−7413	41
H(00K)	−2142	−4821	−5417	41	HC	1193	−6003	−7299	41
H(00L)	−2519	−6847	−6897	39	H(4AA)	1533	−4838	−8166	54
H(00M)	82	−6963	−6794	39	HD	2137	−5720	−7867	54
H(00N)	1812	−4671	−6894	49	HE	1674	−4444	−7767	54
H(00O)	2178	−5384	−6546	49	H(5AA)	−7480	−5097	−4603	60
H(00P)	1083	−4166	−6525	49	HF	−5748	−5625	−4329	60
H(00Q)	−1405	−7544	−6170	63	HG	−6965	−6408	−4610	60
H(00R)	334	−7313	−5859	63	H(6AA)	−5985	−3709	−5202	67
H(00S)	967	−6967	−6250	63	HH	−8126	−4477	−5121	67
H(00T)	−168	−6165	−8542	43	HI	−7204	−4369	−5512	67

Figure S1. 1H-NMR spectrum of penicisteroid D (1)

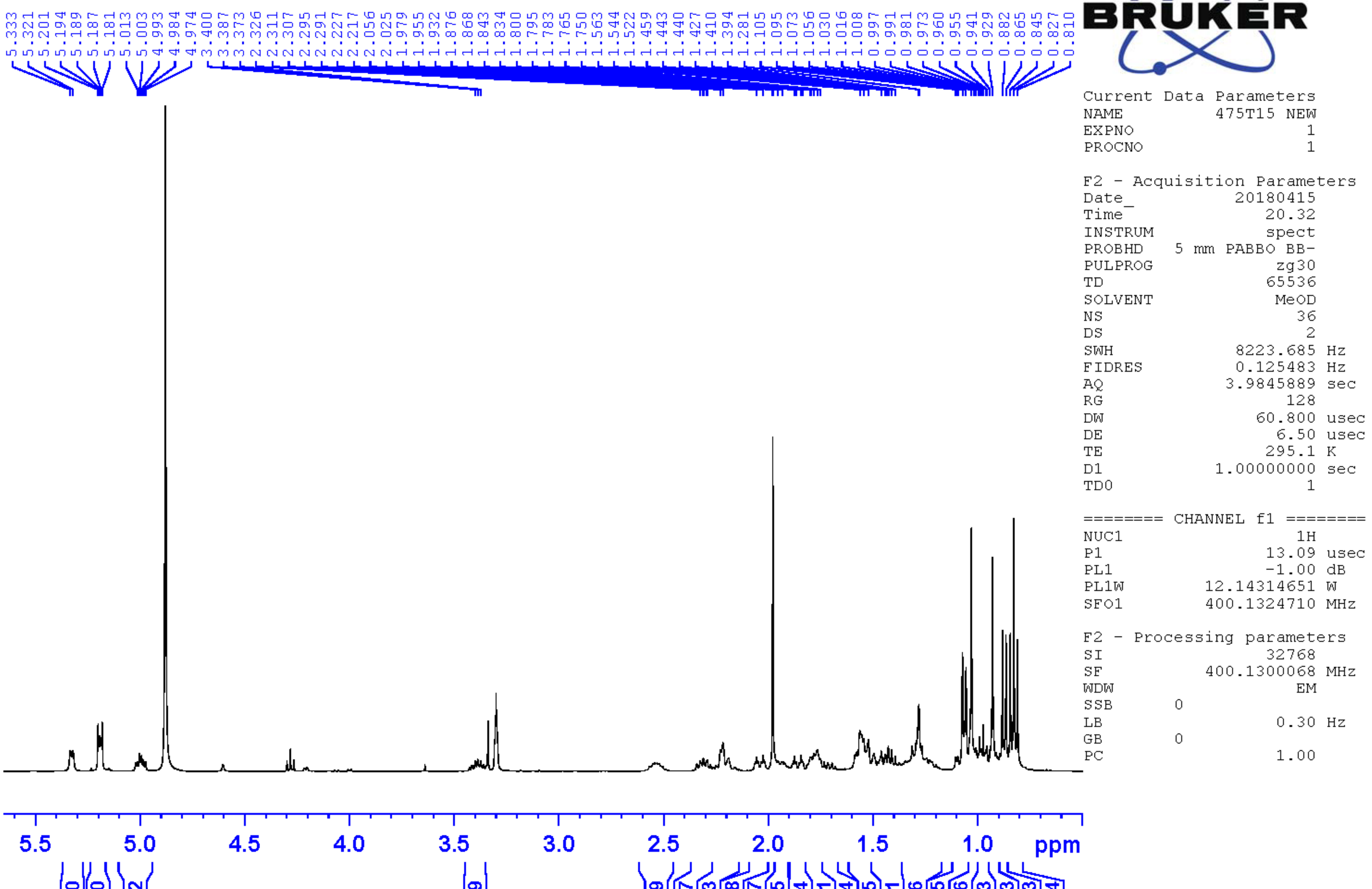

Figure S2. 13C-NMR spectrum of penicisteroid D (1)

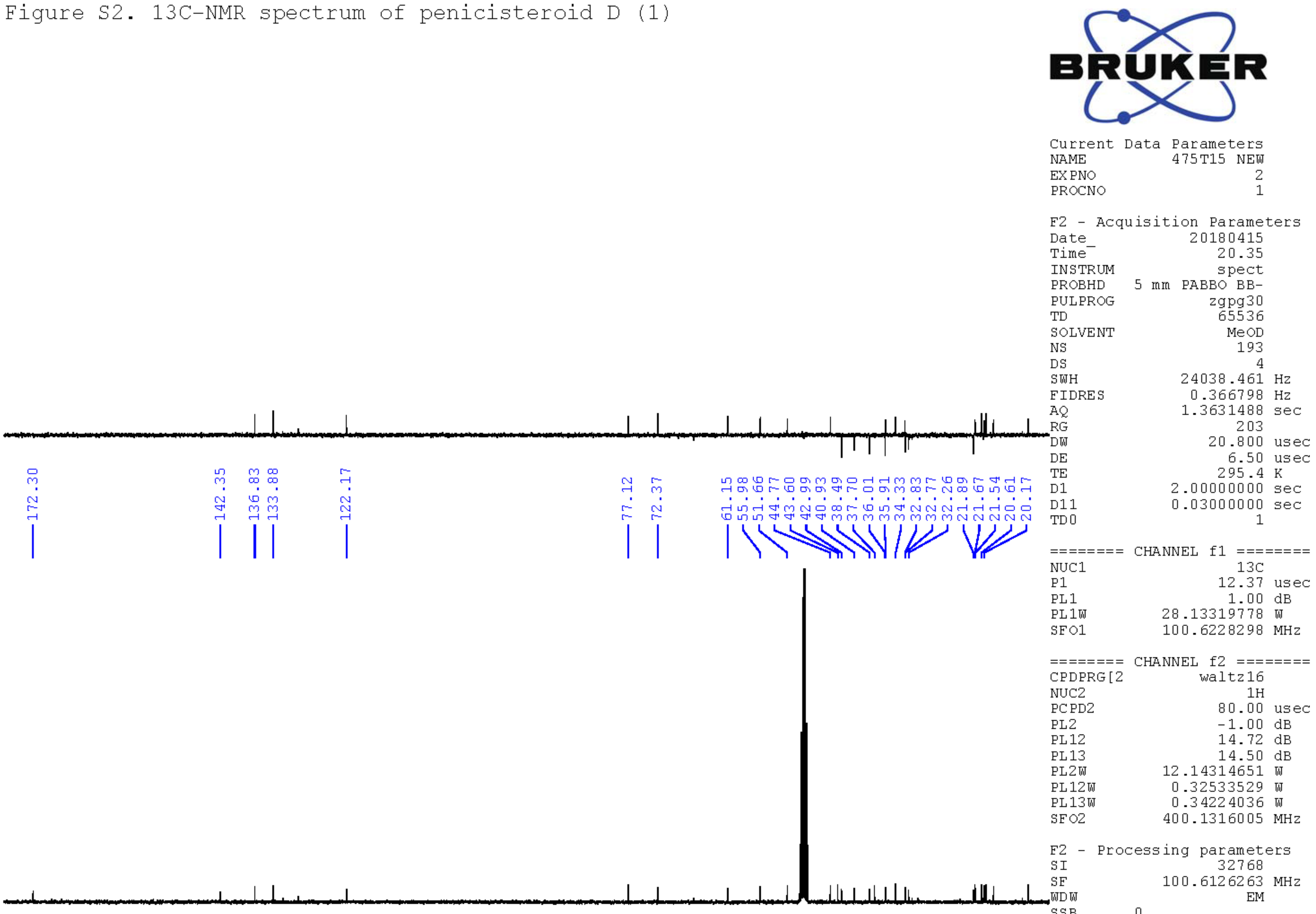

Figure S3. HSQC spectrum of the penicisteroid D (1)

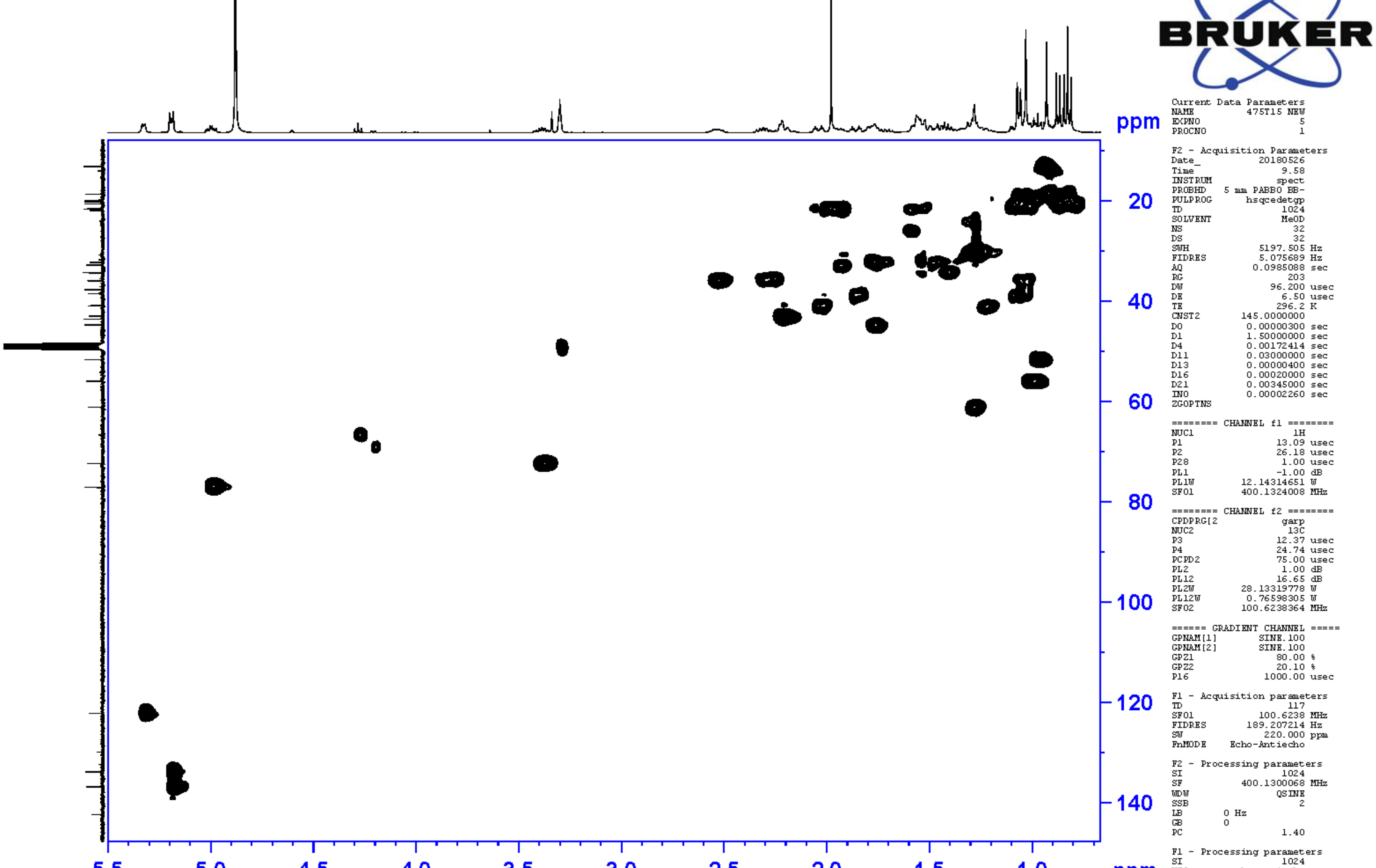

Figure S4. COSY spectrum of penicisteroid D (1)

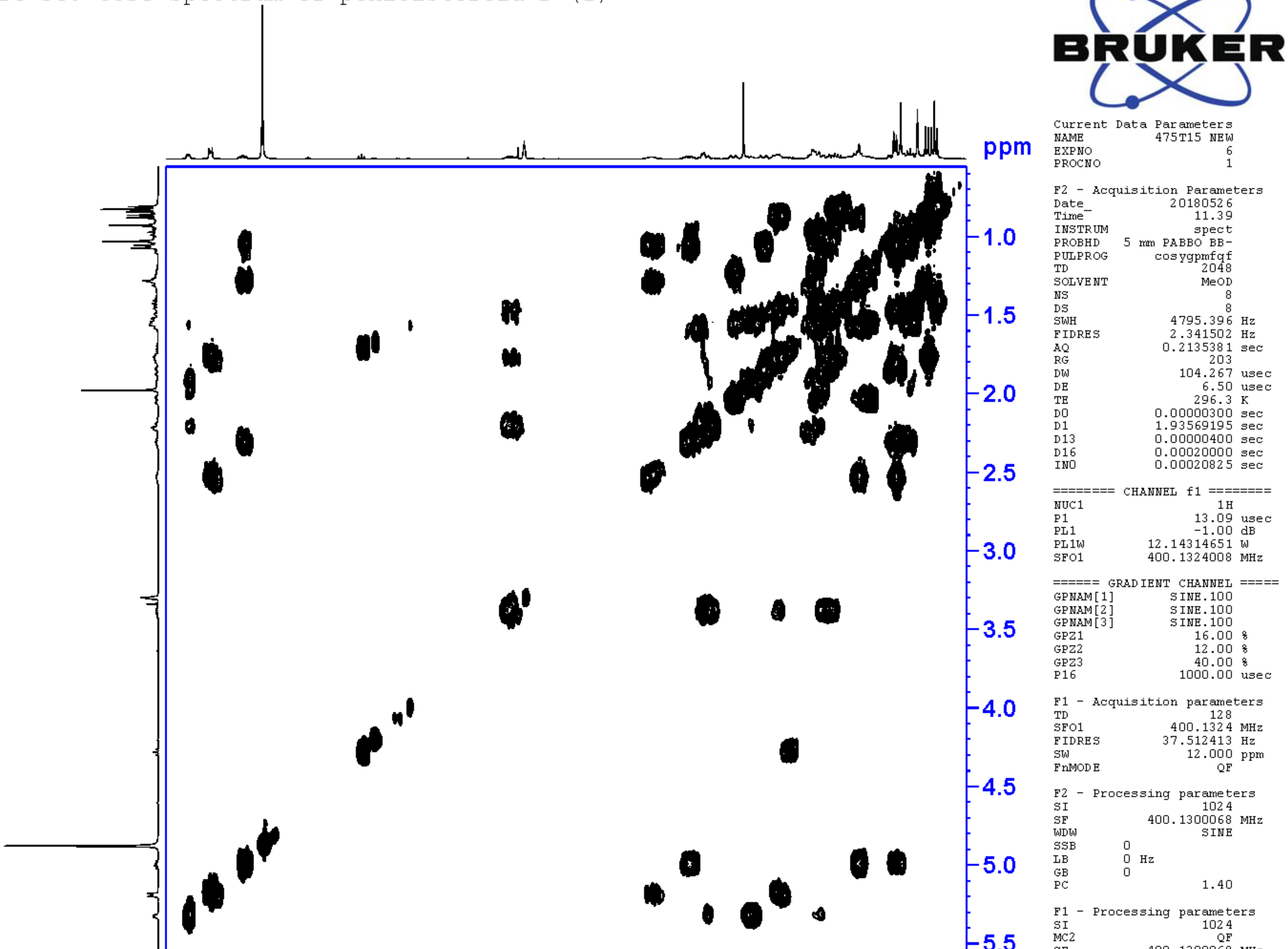

Figure S5. HMBC spectrum of penicisteroid D (1)

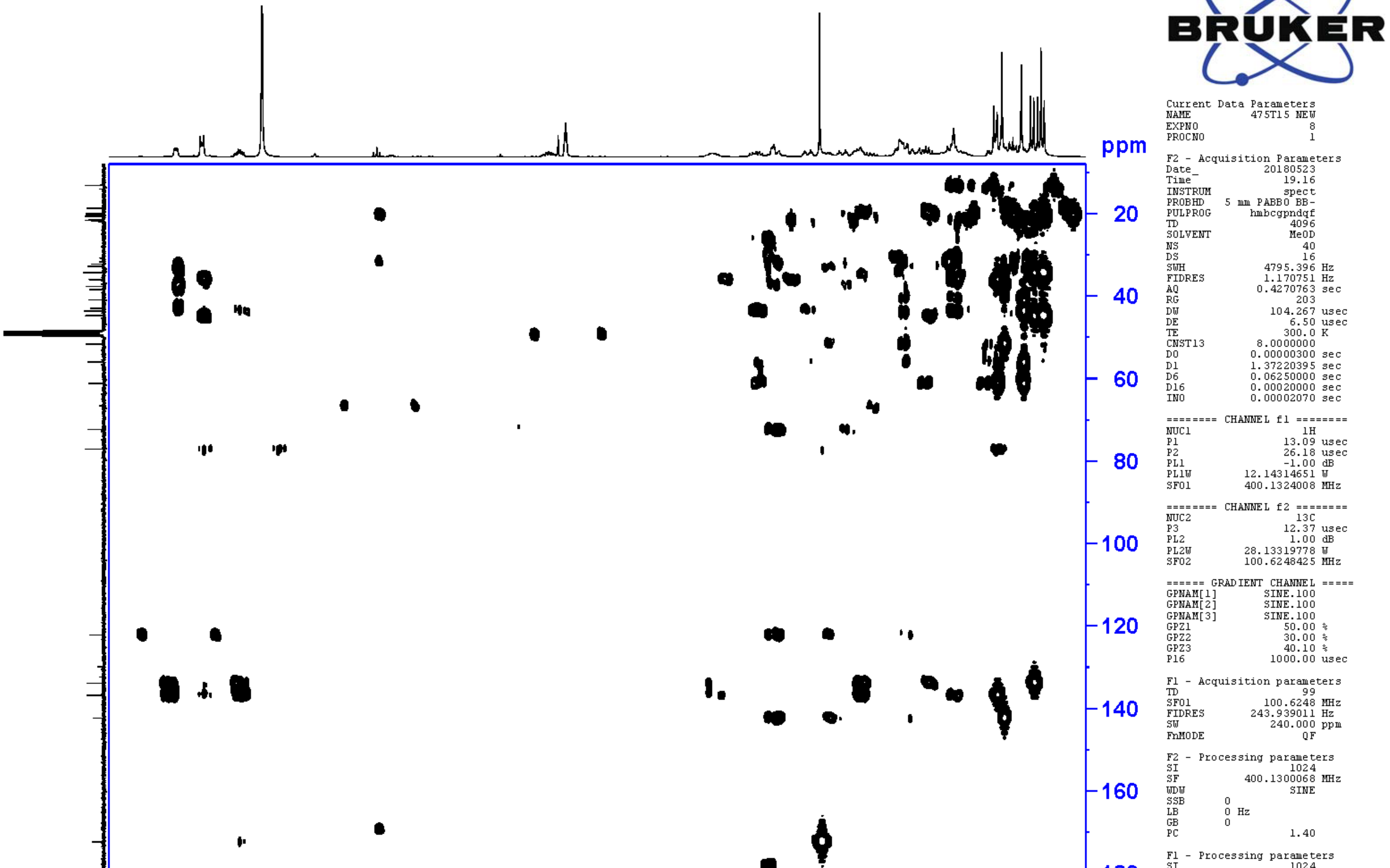

Figure S6. NOESY spectrum of penicisteroid D (1).

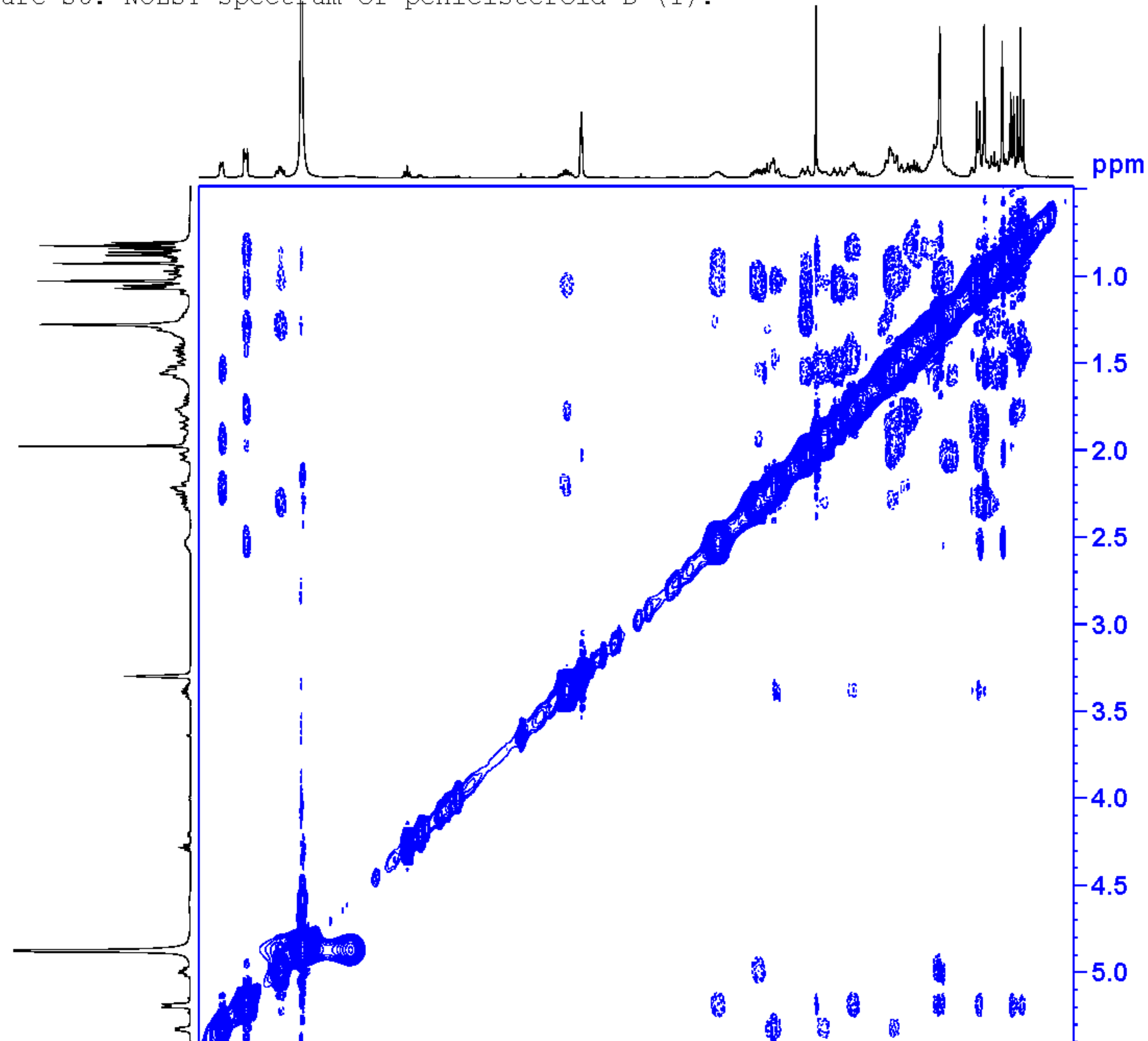


```
Current Data Parameters
NAME            475T15 NEW
EXPNO                    7
PROCNO                   1

F2 - Acquisition Parameters
Date_             20180526
Time                 12.17
INSTRUM              spect
PROBHD   5 mm PABBO BB-
PULPROG            noesyph
TD                    2048
SOLVENT               MeOD
NS                      36
DS                       4
SWH               4795.396 Hz
FIDRES            2.341502 Hz
AQ               0.2135381 sec
RG                     203
DW                 104.267 usec
DE                    6.50 usec
TE                   296.0 K
D0              0.00008747 sec
D1              2.00000000 sec
D8              0.30000001 sec
IN0             0.00020825 sec

======== CHANNEL f1 ========
NUC1                    1H
P1                   13.09 usec
PL1                  -1.00 dB
PL1W           12.14314651 W
SFO1           400.1324008 MHz

F1 - Acquisition parameters
TD                     221
SFO1              400.1324 MHz
FIDRES           21.726646 Hz
SW                  12.000 ppm
FnMODE         States-TPPI

F2 - Processing parameters
SI                    1024
SF             400.1300068 MHz
WDW                  QSINE
SSB                      2
LB         0 Hz
GB         0
PC                    1.00

F1 - Processing parameters
SI                    1024
MC2            States-TPPI
SF             400.1300068 MHz
```

Figure S7. 1H-NMR spectrum of penicisteroid E (2)

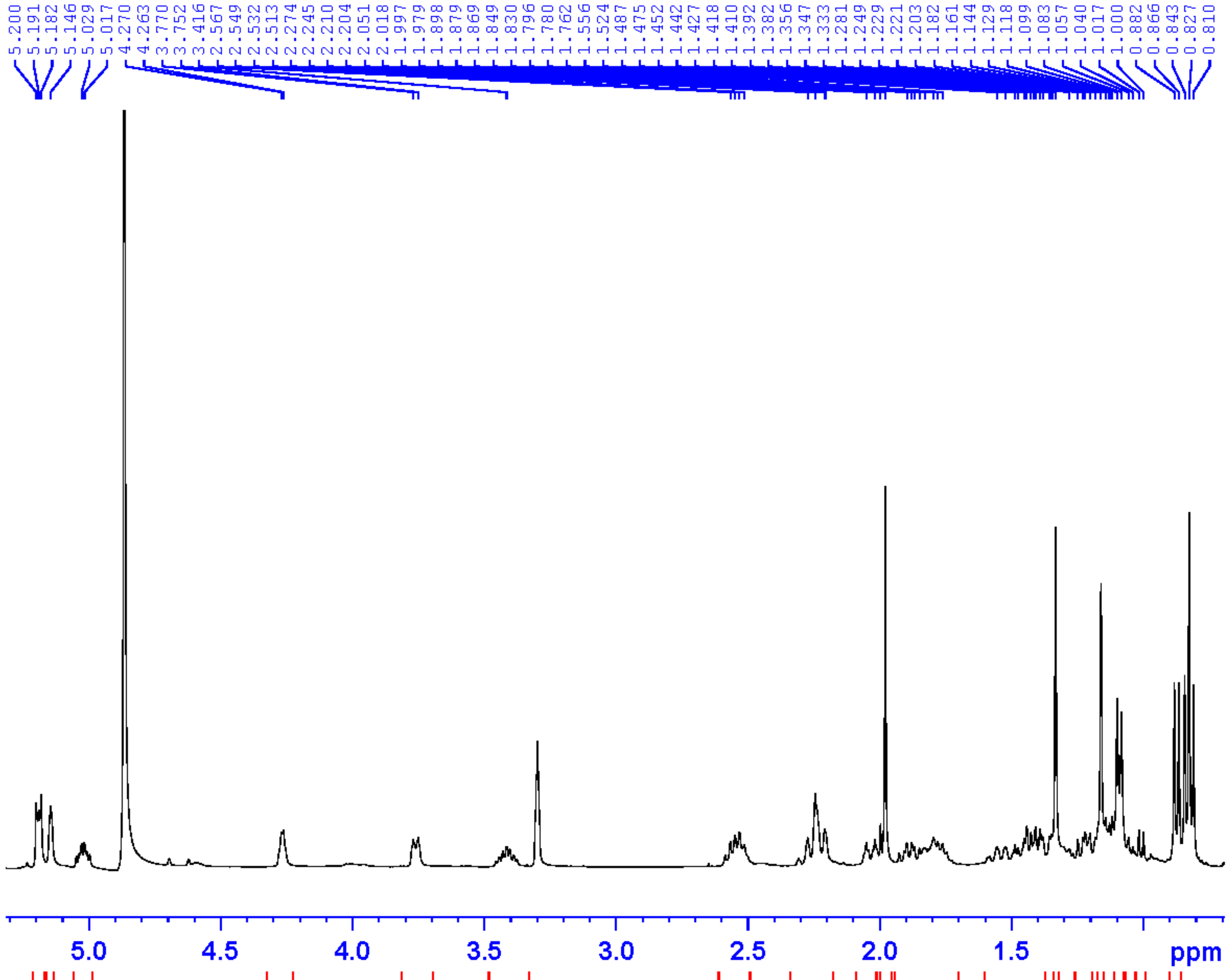


```
Current Data Parameters
NAME            475T51 NEW
EXPNO                    1
PROCNO                   1

F2 - Acquisition Parameters
Date_             20180509
Time                 17.34
INSTRUM              spect
PROBHD    5 mm PABBO BB-
PULPROG               zg30
TD                   65536
SOLVENT               MeOD
NS                      35
DS                       2
SWH               8223.685 Hz
FIDRES            0.125483 Hz
AQ               3.9845889 sec
RG                     128
DW                  60.800 usec
DE                    6.50 usec
TE                   296.5 K
D1              1.00000000 sec
TD0                      1

======== CHANNEL f1 ========
NUC1                    1H
P1                   13.09 usec
PL1                  -1.00 dB
PL1W           12.14314651 W
SFO1           400.1324710 MHz

F2 - Processing parameters
SI                   32768
SF             400.1300068 MHz
WDW                     EM
SSB      0
LB                    0.30 Hz
GB       0
PC                    1.00
```

Figure S8. 13C-NMR spectrum of penicisteroid E (2)

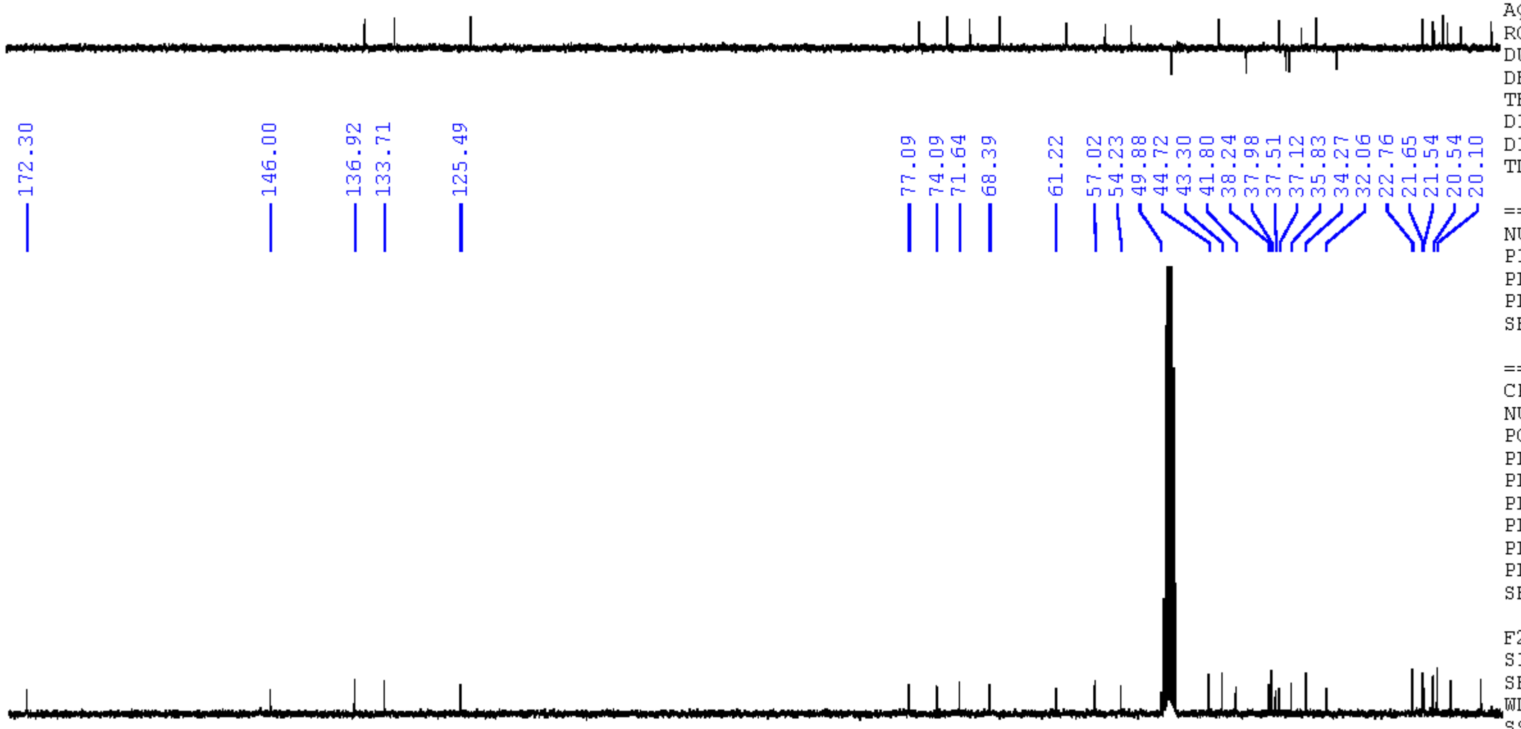

Current Data Parameters
NAME 475T51 NEW
EXPNO 2
PROCNO 1

F2 - Acquisition Parameters
Date_ 20180509
Time 17.38
INSTRUM spect
PROBHD 5 mm PABBO BB-
PULPROG zgpg30
TD 65536
SOLVENT MeOD
NS 276
DS 4
SWH 24038.461 Hz
FIDRES 0.366798 Hz
AQ 1.3631488 sec
RG 203
DW 20.800 usec
DE 6.50 usec
TE 296.5 K
D1 2.00000000 sec
D11 0.03000000 sec
TD0 1

======== CHANNEL f1 ========
NUC1 13C
P1 12.37 usec
PL1 1.00 dB
PL1W 28.13319778 W
SFO1 100.6228298 MHz

======== CHANNEL f2 ========
CPDPRG[2 waltz16
NUC2 1H
PCPD2 80.00 usec
PL2 -1.00 dB
PL12 14.72 dB
PL13 14.50 dB
PL2W 12.14314651 W
PL12W 0.32533529 W
PL13W 0.34224036 W
SFO2 400.1316005 MHz

F2 - Processing parameters
SI 32768
SF 100.6126271 MHz
WDW EM
SSB 0

Figure S9. HSQC spectrum of penicisteroid E (2)

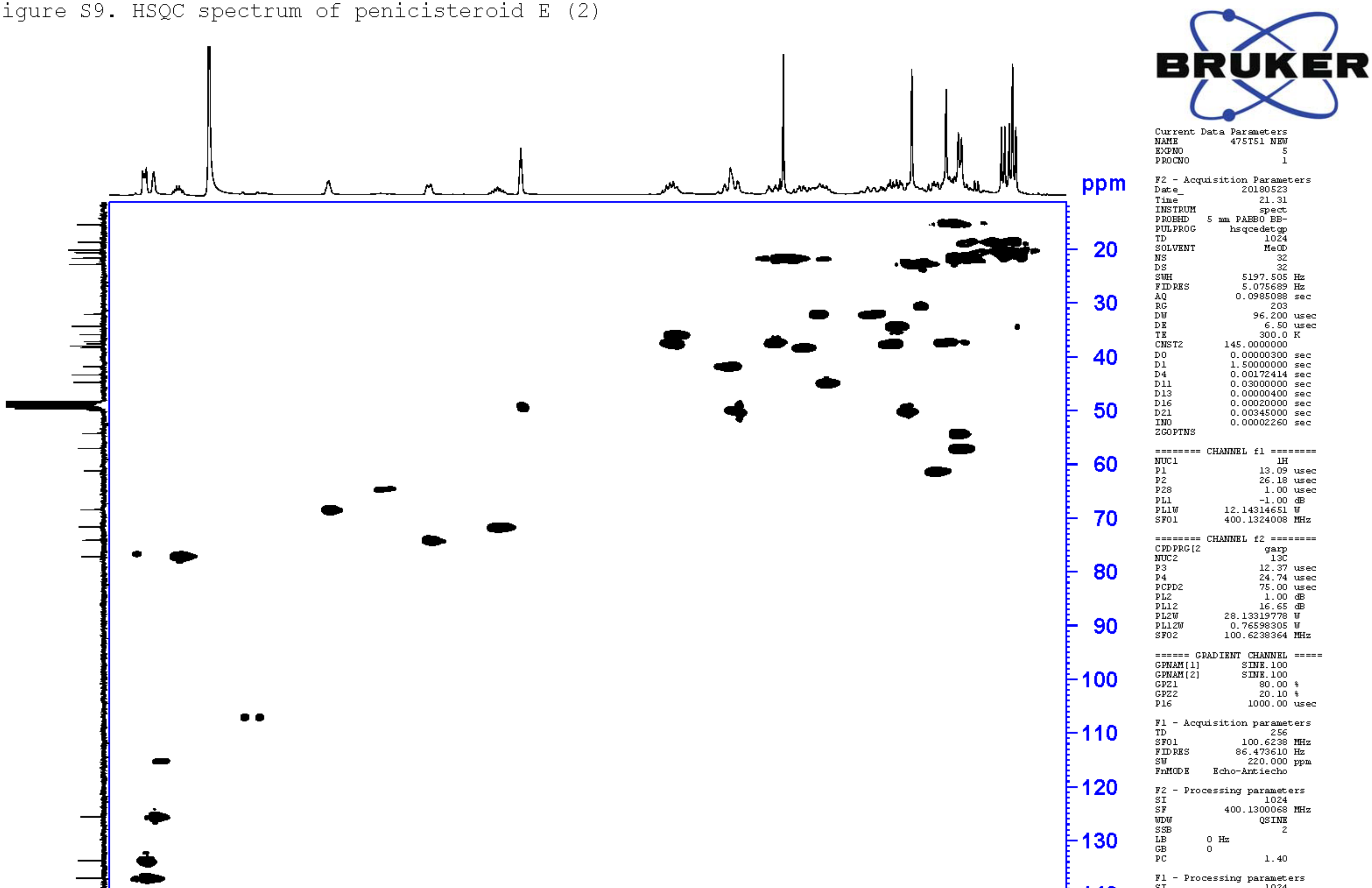

Figure S10. COSY spectrum of penicisteroid E (2)

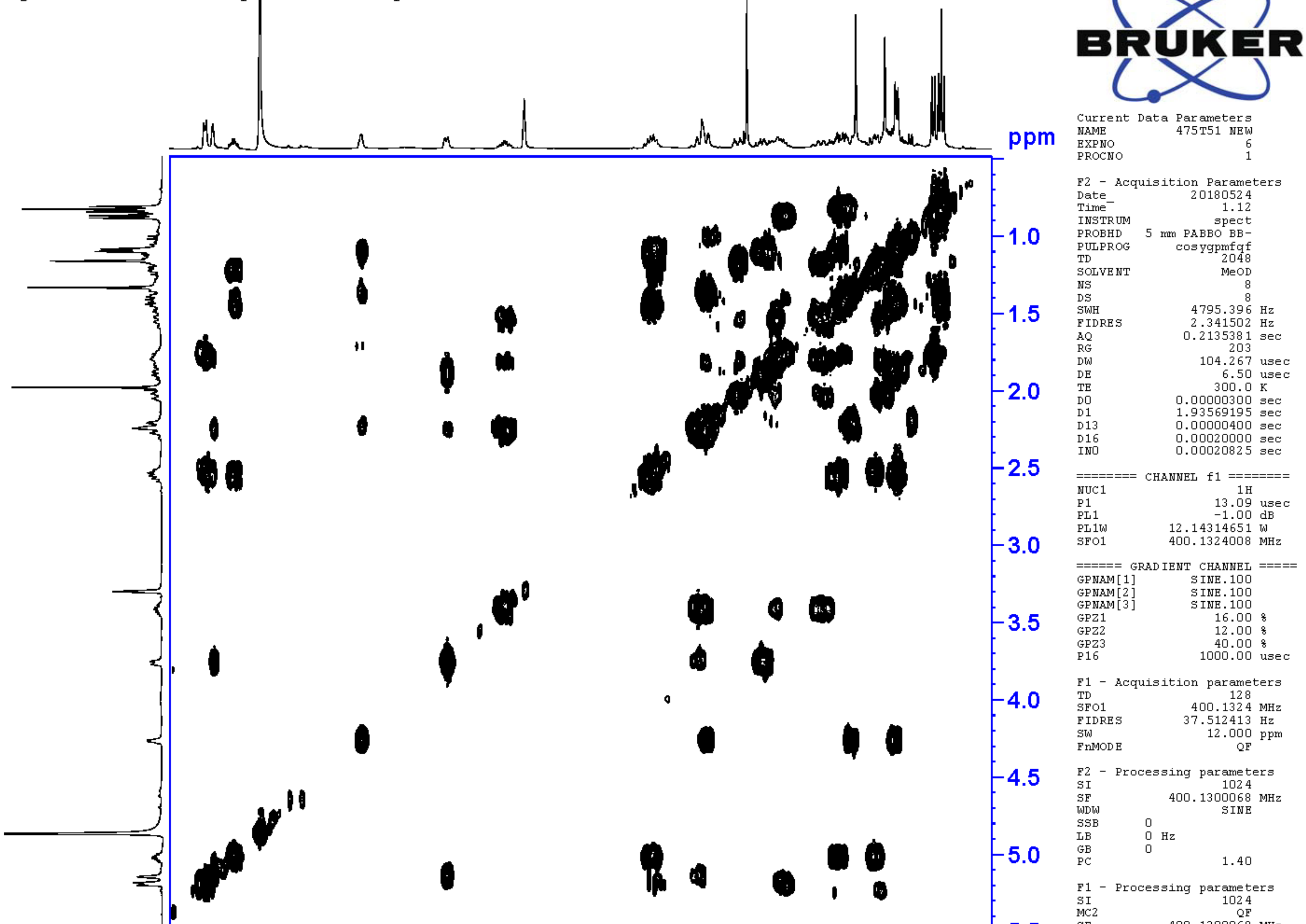

Figure S11. HMBC spectrum of penicisteroid E (2)

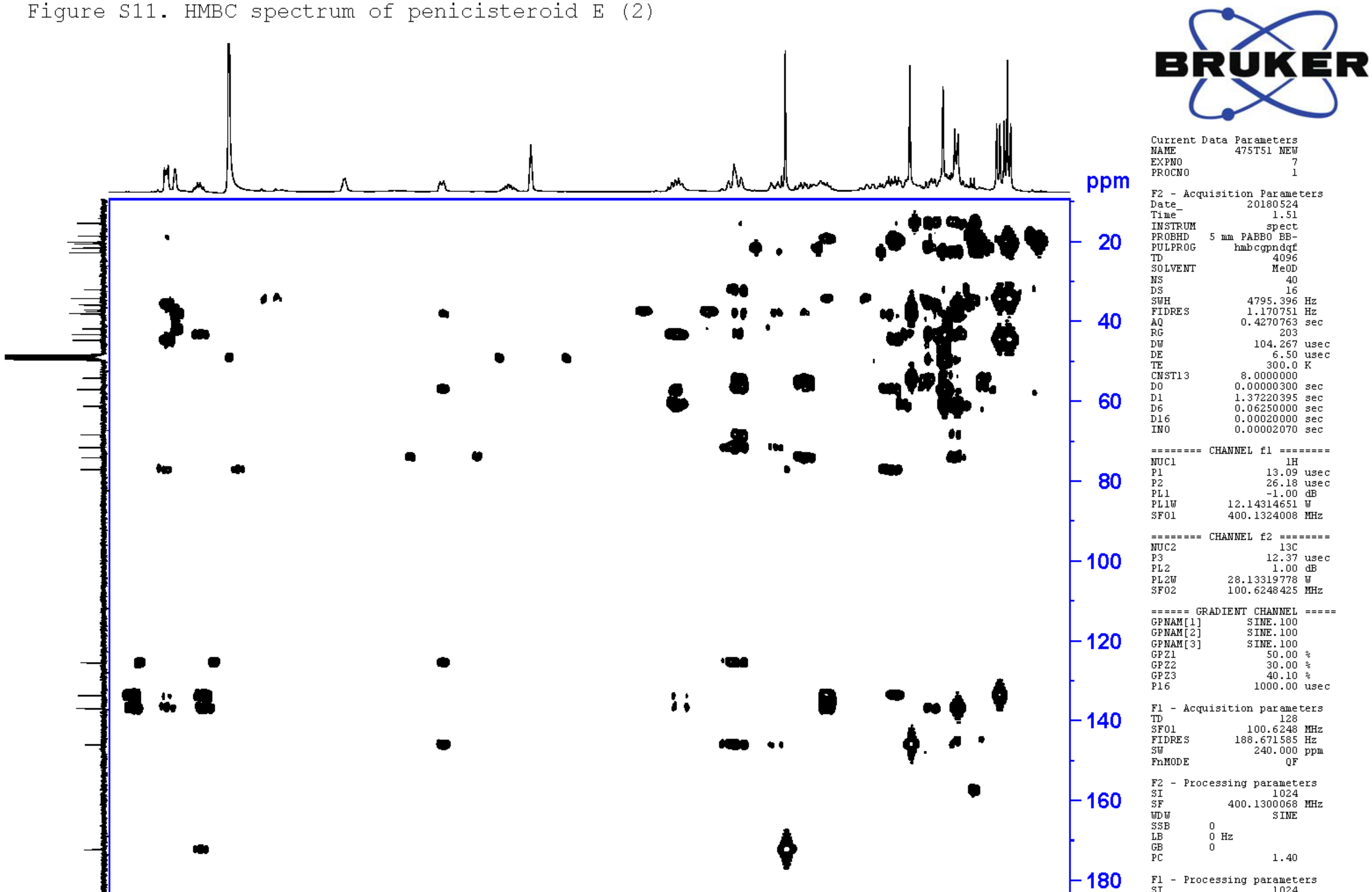

Figure S12. NOESY spectrum of penicisteroid E (2).

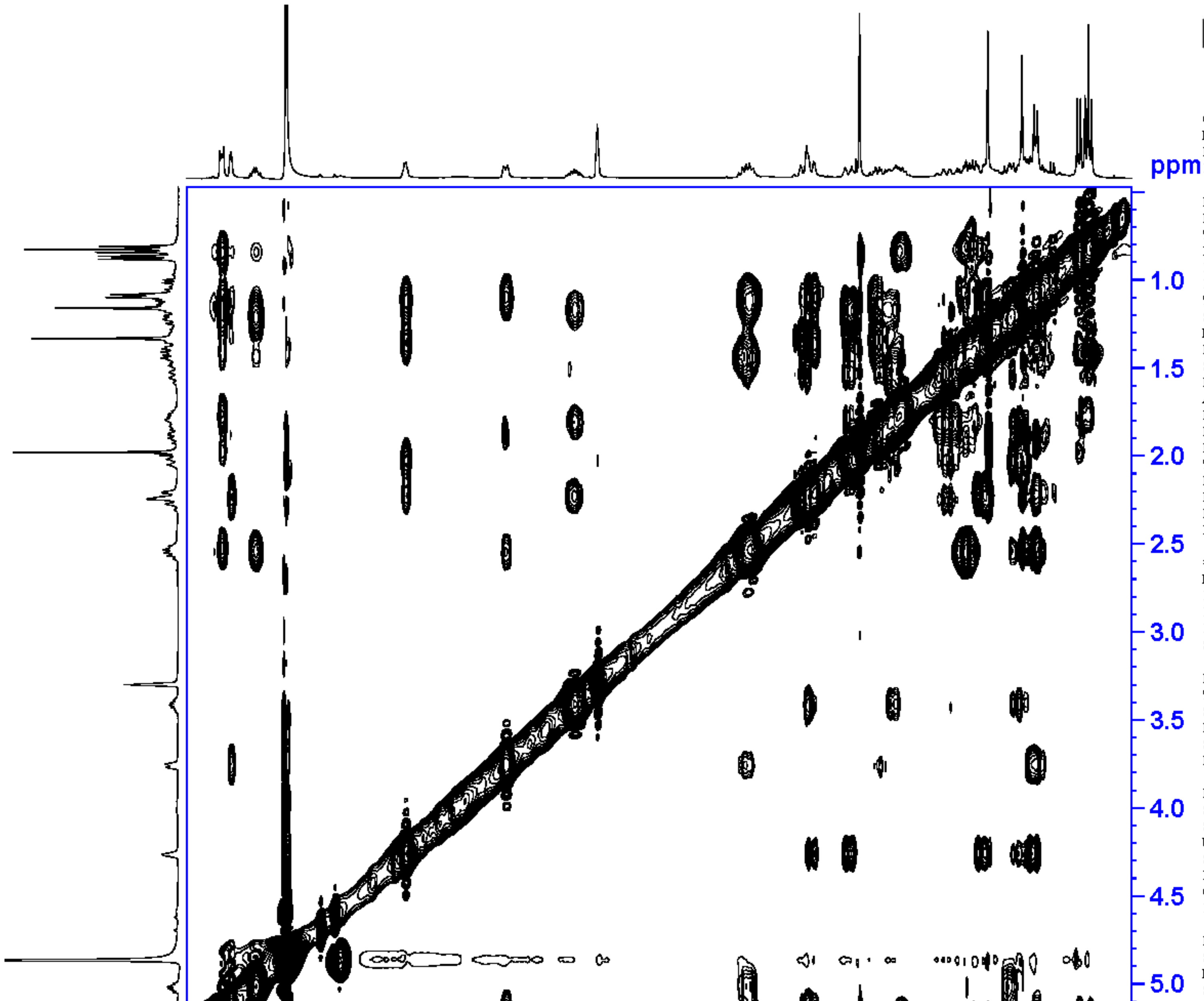

Current Data Parameters
NAME 475T51 NEW
EXPNO 8
PROCNO 1

F2 - Acquisition Parameters
Date_ 20180524
Time 4.31
INSTRUM spect
PROBHD 5 mm PABBO BB-
PULPROG noesyph
TD 2048
SOLVENT MeOD
NS 32
DS 4
SWH 4795.396 Hz
FIDRES 2.341502 Hz
AQ 0.2135381 sec
RG 203
DW 104.267 usec
DE 6.50 usec
TE 300.0 K
D0 0.00008747 sec
D1 2.00000000 sec
D8 0.30000001 sec
IN0 0.00020825 sec

======== CHANNEL f1 ========
NUC1 1H
P1 13.09 usec
PL1 -1.00 dB
PL1W 12.14314651 W
SFO1 400.1324008 MHz

F1 - Acquisition parameters
TD 174
SFO1 400.1324 MHz
FIDRES 27.595339 Hz
SW 12.000 ppm
FnMODE States-TPPI

F2 - Processing parameters
SI 1024
SF 400.1300068 MHz
WDW QSINE
SSB 2
LB 0 Hz
GB 0
PC 1.00

F1 - Processing parameters
SI 1024
MC2 States-TPPI
SF 400.1300068 MHz

Figure S13. 1H-NMR spectrum of penicisteroid F (3)

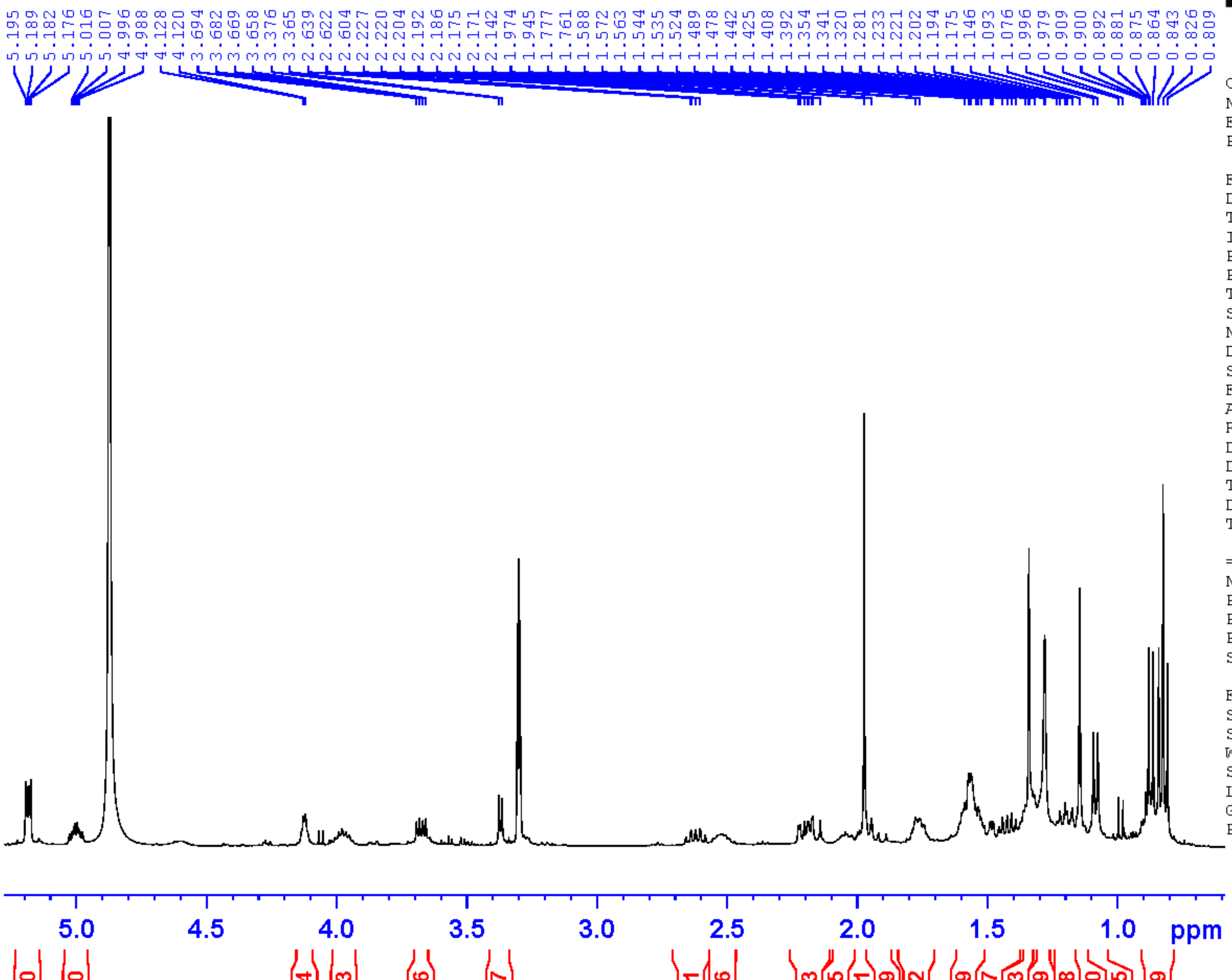


```
Current Data Parameters
NAME              475T2 NEW
EXPNO                     1
PROCNO                    1

F2 - Acquisition Parameters
Date_              20180328
Time                  22.29
INSTRUM               spect
PROBHD    5 mm PABBO BB-
PULPROG                zg30
TD                    65536
SOLVENT                MeOD
NS                      128
DS                        2
SWH                8223.685 Hz
FIDRES             0.125483 Hz
AQ                3.9845889 sec
RG                      203
DW                   60.800 usec
DE                     6.50 usec
TE                    296.0 K
D1               1.00000000 sec
TD0                       1

======== CHANNEL f1 ========
NUC1                     1H
P1                    13.09 usec
PL1                   -1.00 dB
PL1W            12.14314651 W
SFO1            400.1324710 MHz

F2 - Processing parameters
SI                    32768
SF              400.1300068 MHz
WDW                      EM
SSB       0
LB                     0.30 Hz
GB        0
PC                     1.00
```

Figure S14. 13C-NMR spectrum of penicisteroid F (3)

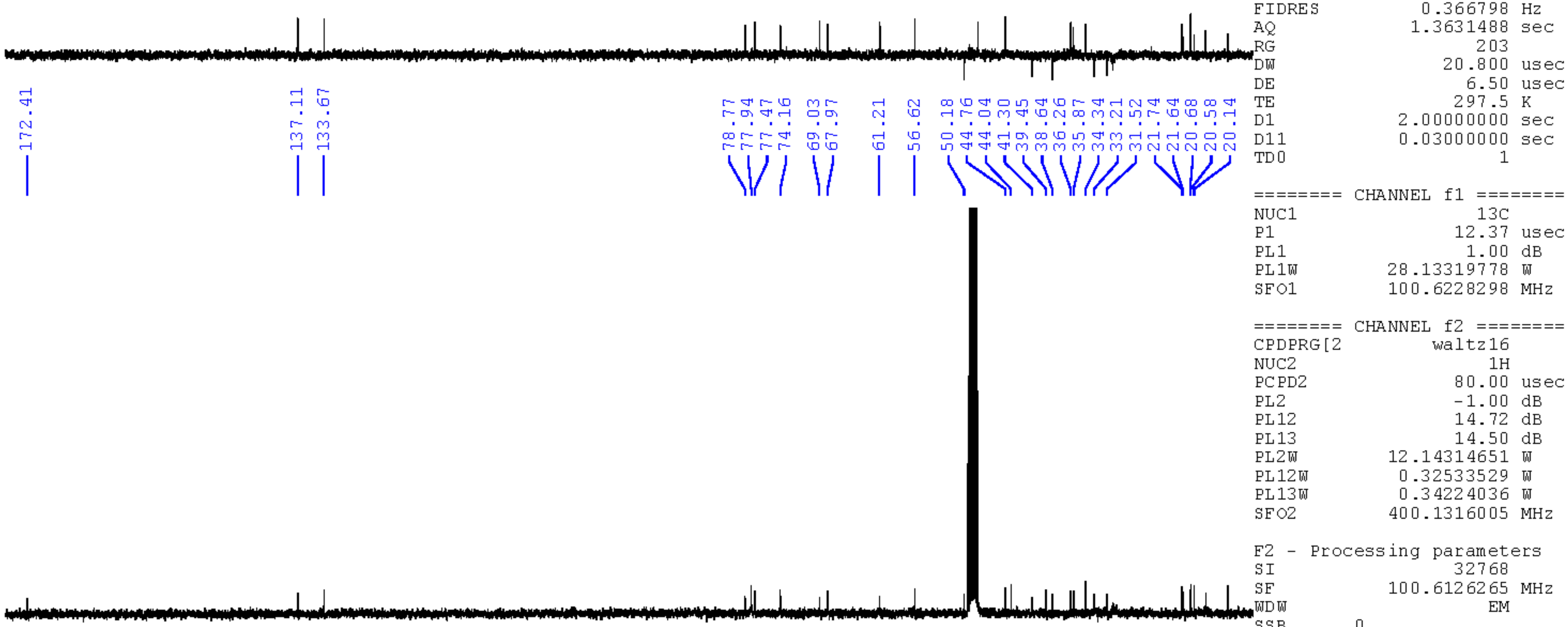


```
Current Data Parameters
NAME          475T2 NEW
EXPNO                 2
PROCNO                1

F2 - Acquisition Parameters
Date_          20180329
Time               3.16
INSTRUM           spect
PROBHD   5 mm PABBO BB-
PULPROG         zgpg30
TD               65536
SOLVENT            MeOD
NS                 5012
DS                    4
SWH           24038.461 Hz
FIDRES         0.366798 Hz
AQ            1.3631488 sec
RG                  203
DW               20.800 usec
DE                 6.50 usec
TE                297.5 K
D1           2.00000000 sec
D11          0.03000000 sec
TD0                   1

======== CHANNEL f1 ========
NUC1                13C
P1                12.37 usec
PL1                1.00 dB
PL1W        28.13319778 W
SFO1        100.6228298 MHz

======== CHANNEL f2 ========
CPDPRG[2        waltz16
NUC2                 1H
PCPD2             80.00 usec
PL2               -1.00 dB
PL12              14.72 dB
PL13              14.50 dB
PL2W        12.14314651 W
PL12W        0.32533529 W
PL13W        0.34224036 W
SFO2        400.1316005 MHz

F2 - Processing parameters
SI                32768
SF          100.6126265 MHz
WDW                  EM
SSB       0
```

Figure S15. HSQC spectrum of penicisteroid F (3)

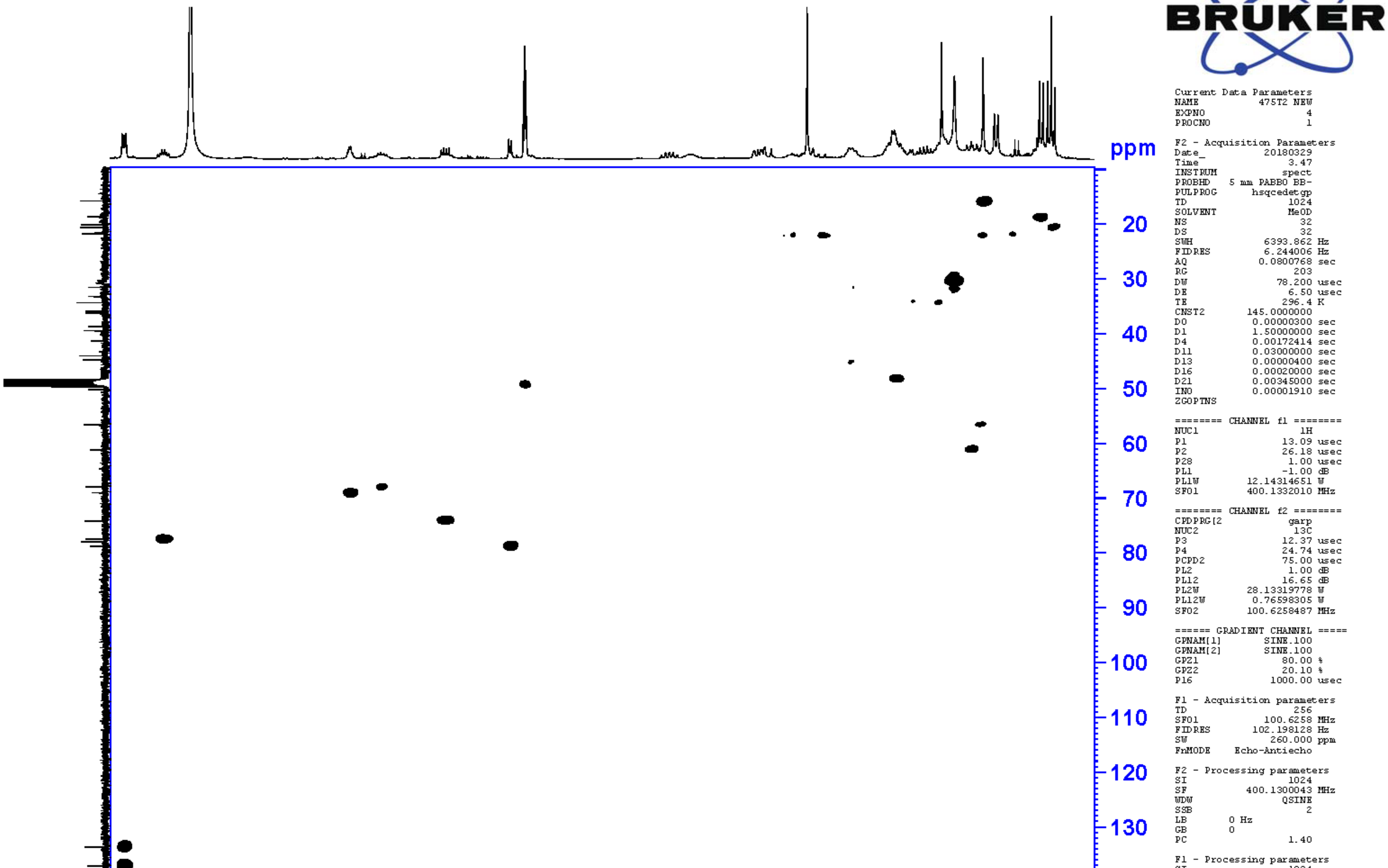

Figure S16. COSY spectrum of penicisteroid F (3)

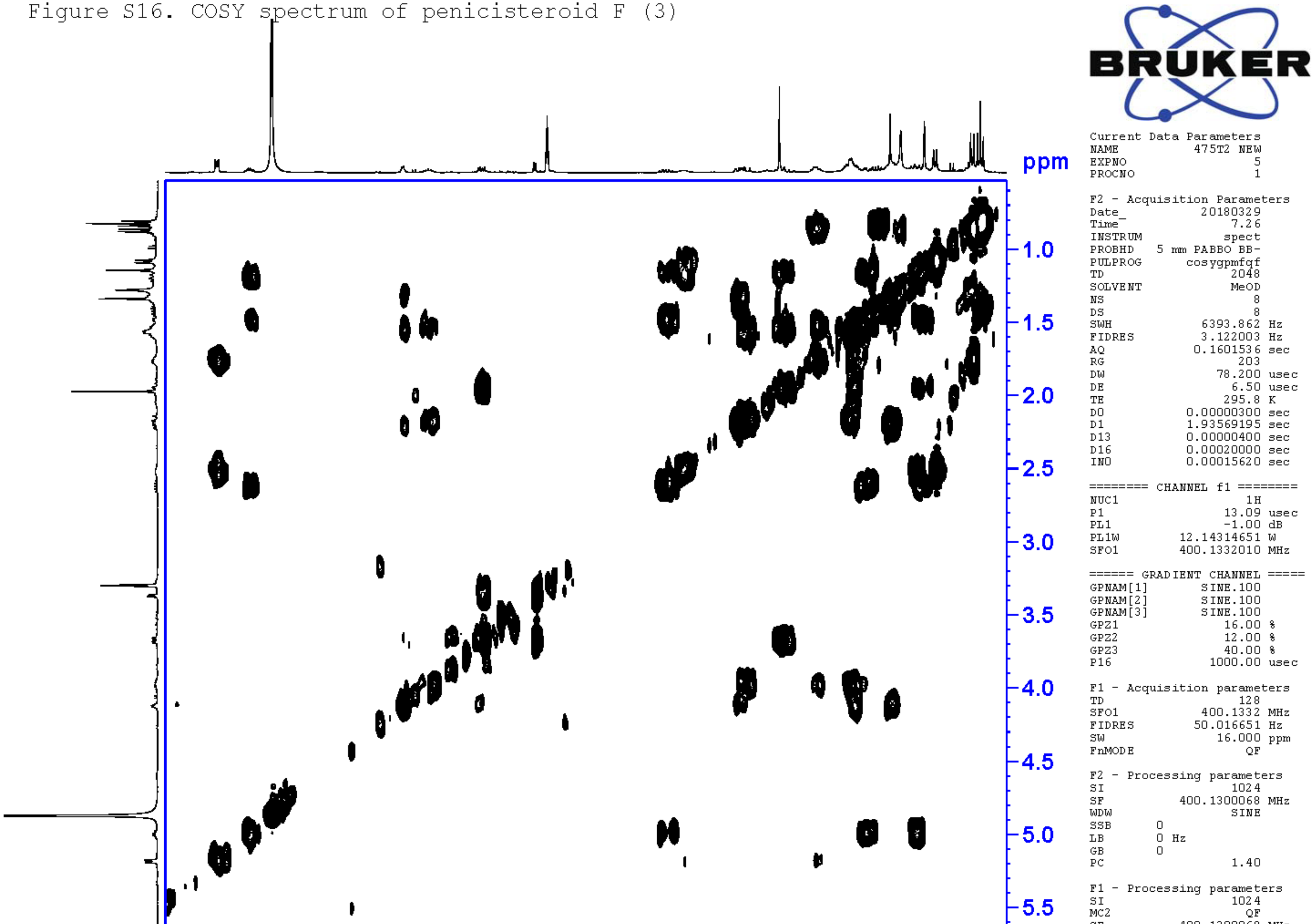

Figure S17. HMBC spectrum of penicisteroid F (3)

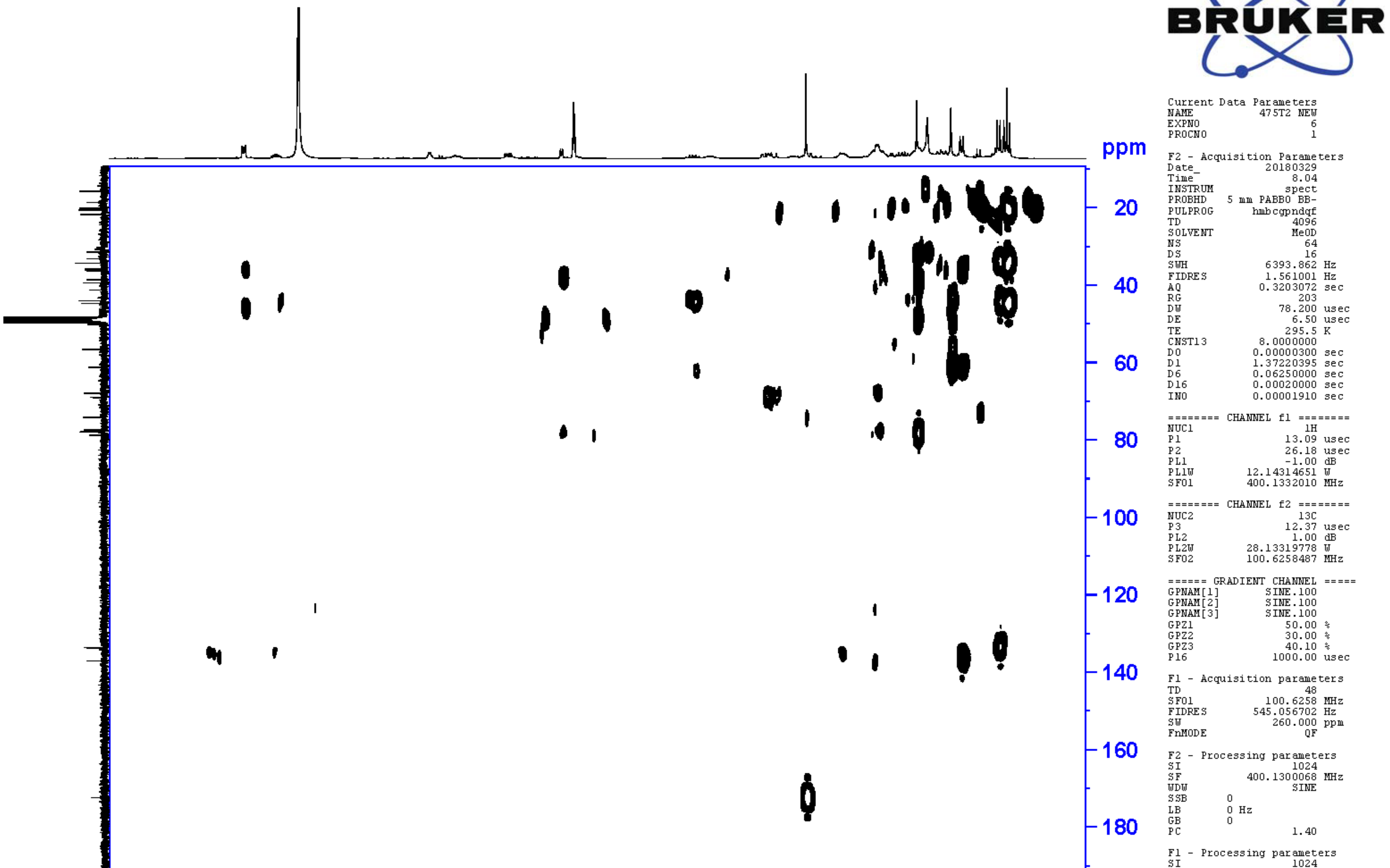

Figure S18. NOESY spectrum of penicisteroid F (3).

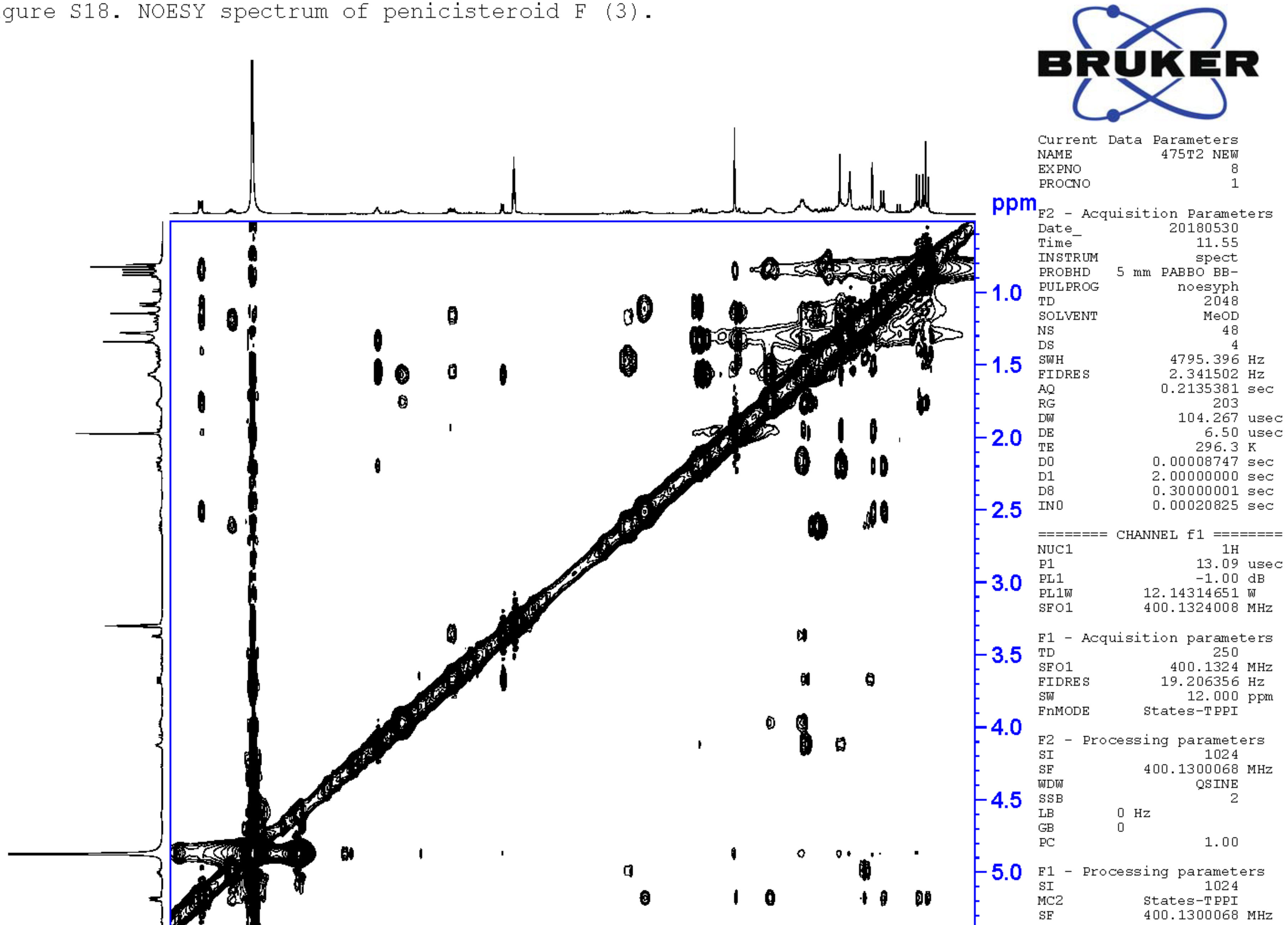

Figure S19. 1H-NMR spectrum of penicisteroid G (4)

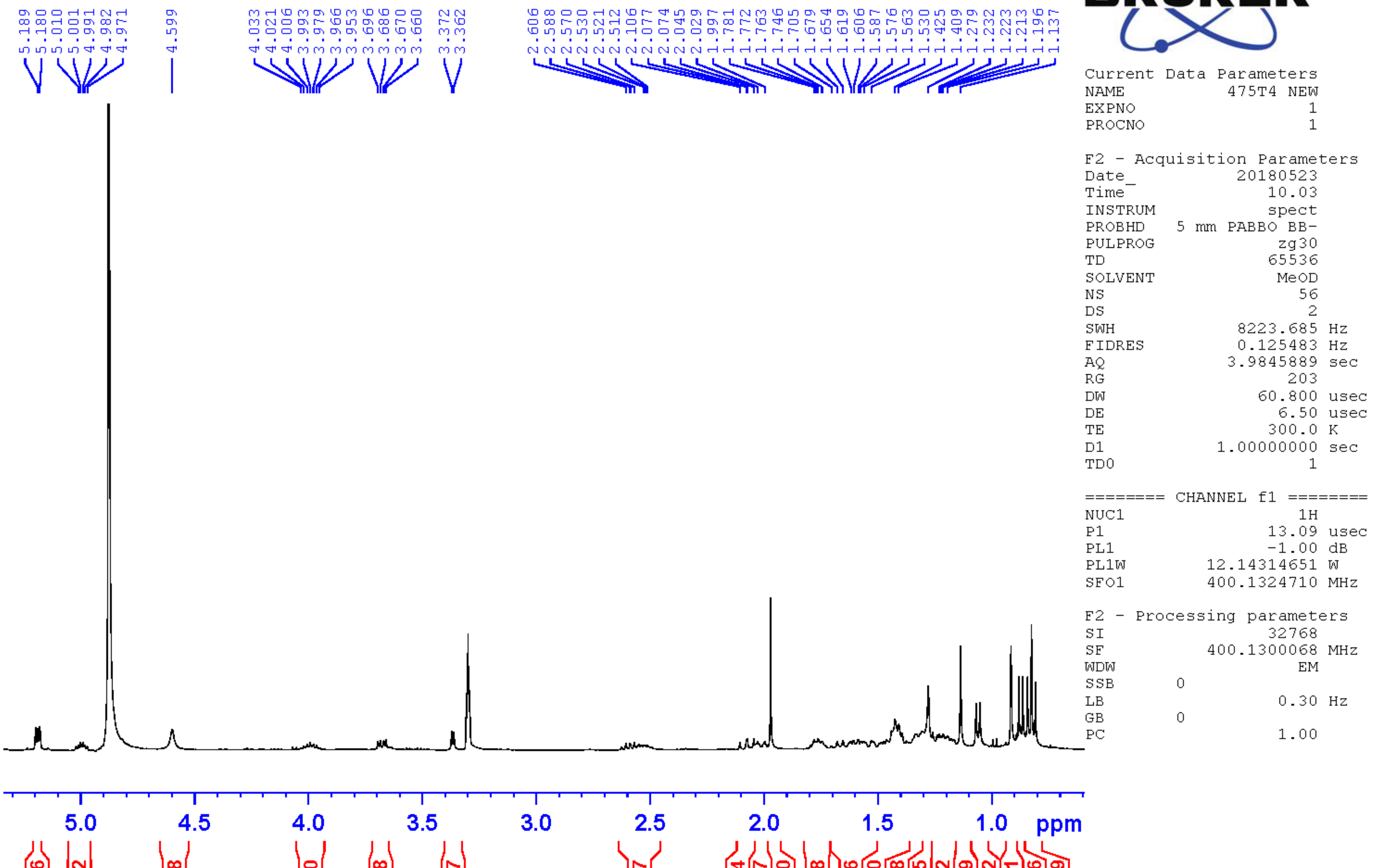

Figure S20. 13C-NMR spectrum of penicisteroid G (4)

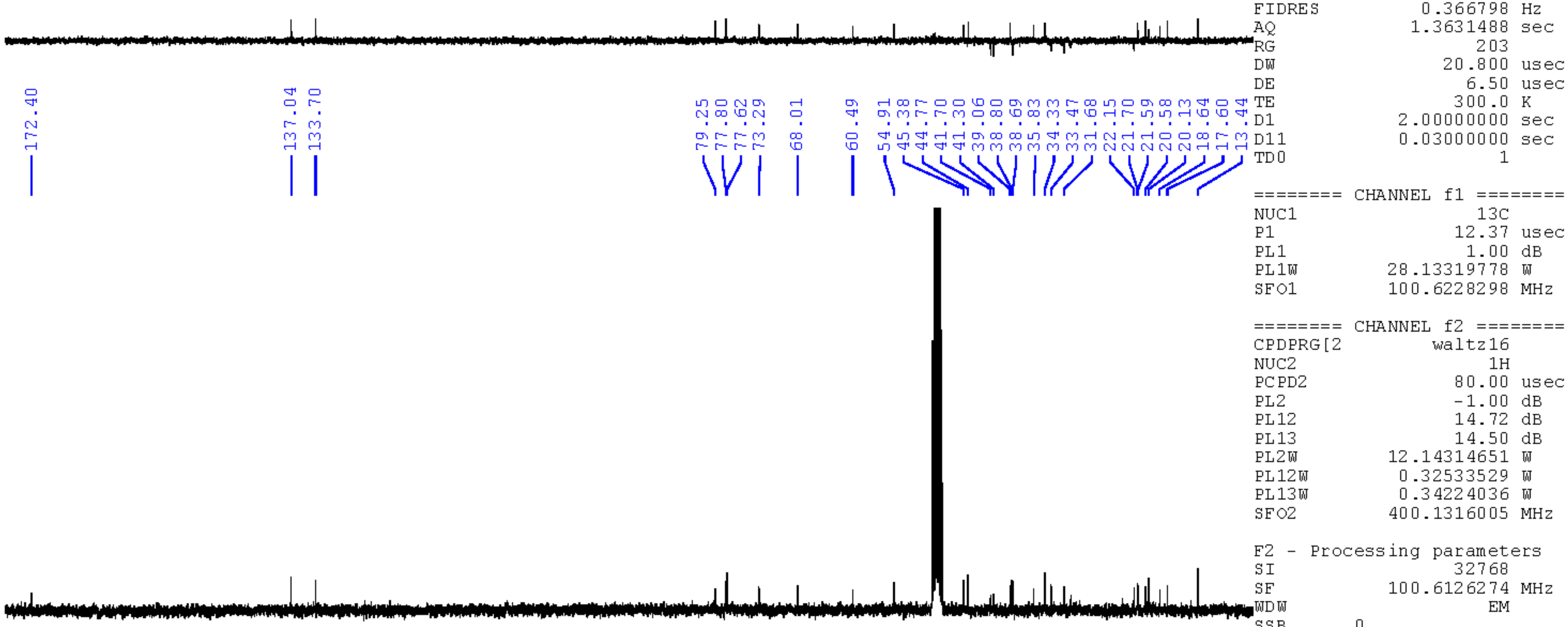

Current Data Parameters
NAME 475T4 NEW
EXPNO 2
PROCNO 1

F2 - Acquisition Parameters
Date_ 20180523
Time 10.09
INSTRUM spect
PROBHD 5 mm PABBO BB-
PULPROG zgpg30
TD 65536
SOLVENT MeOD
NS 5012
DS 4
SWH 24038.461 Hz
FIDRES 0.366798 Hz
AQ 1.3631488 sec
RG 203
DW 20.800 usec
DE 6.50 usec
TE 300.0 K
D1 2.00000000 sec
D11 0.03000000 sec
TD0 1

======== CHANNEL f1 ========
NUC1 13C
P1 12.37 usec
PL1 1.00 dB
PL1W 28.13319778 W
SFO1 100.6228298 MHz

======== CHANNEL f2 ========
CPDPRG[2 waltz16
NUC2 1H
PCPD2 80.00 usec
PL2 -1.00 dB
PL12 14.72 dB
PL13 14.50 dB
PL2W 12.14314651 W
PL12W 0.32533529 W
PL13W 0.34224036 W
SFO2 400.1316005 MHz

F2 - Processing parameters
SI 32768
SF 100.6126274 MHz
WDW EM
SSB 0

Figure S21. HSQC spectrum of penicisteroid G (4)

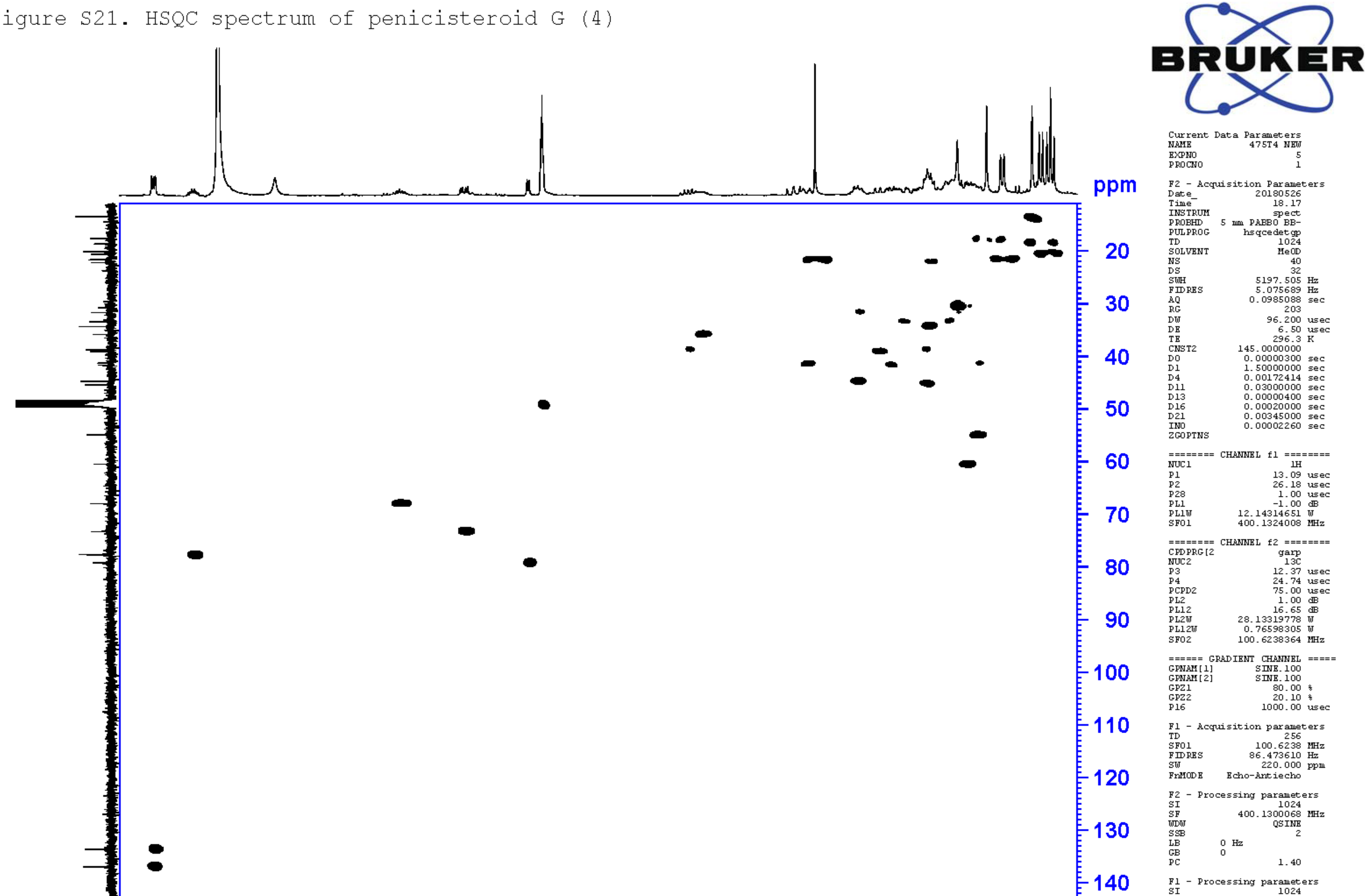

Figure S22. COSY spectrum of penicisteroid G (4)

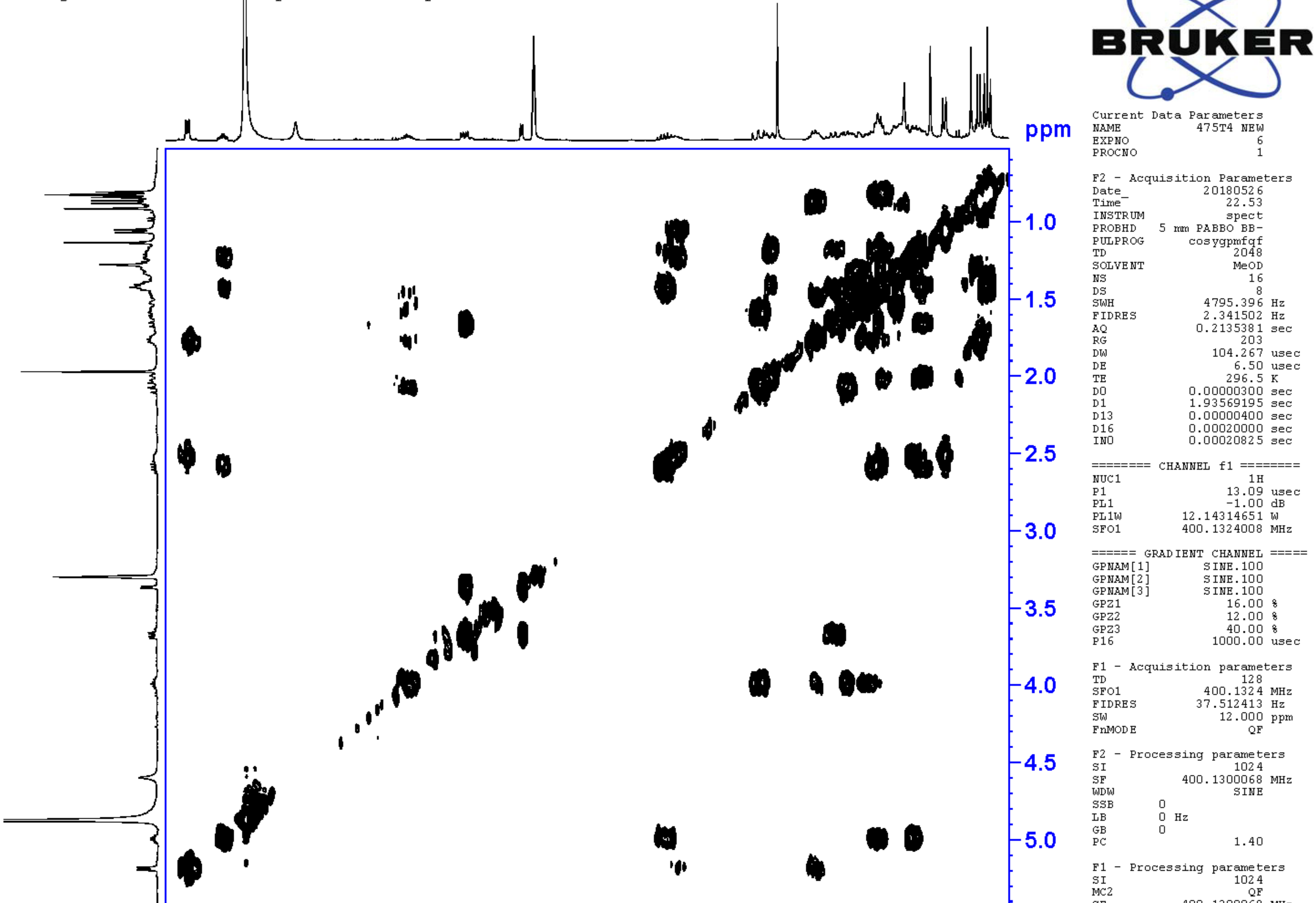

Figure S23. HMBC spectrum of penicisteroid G (4)

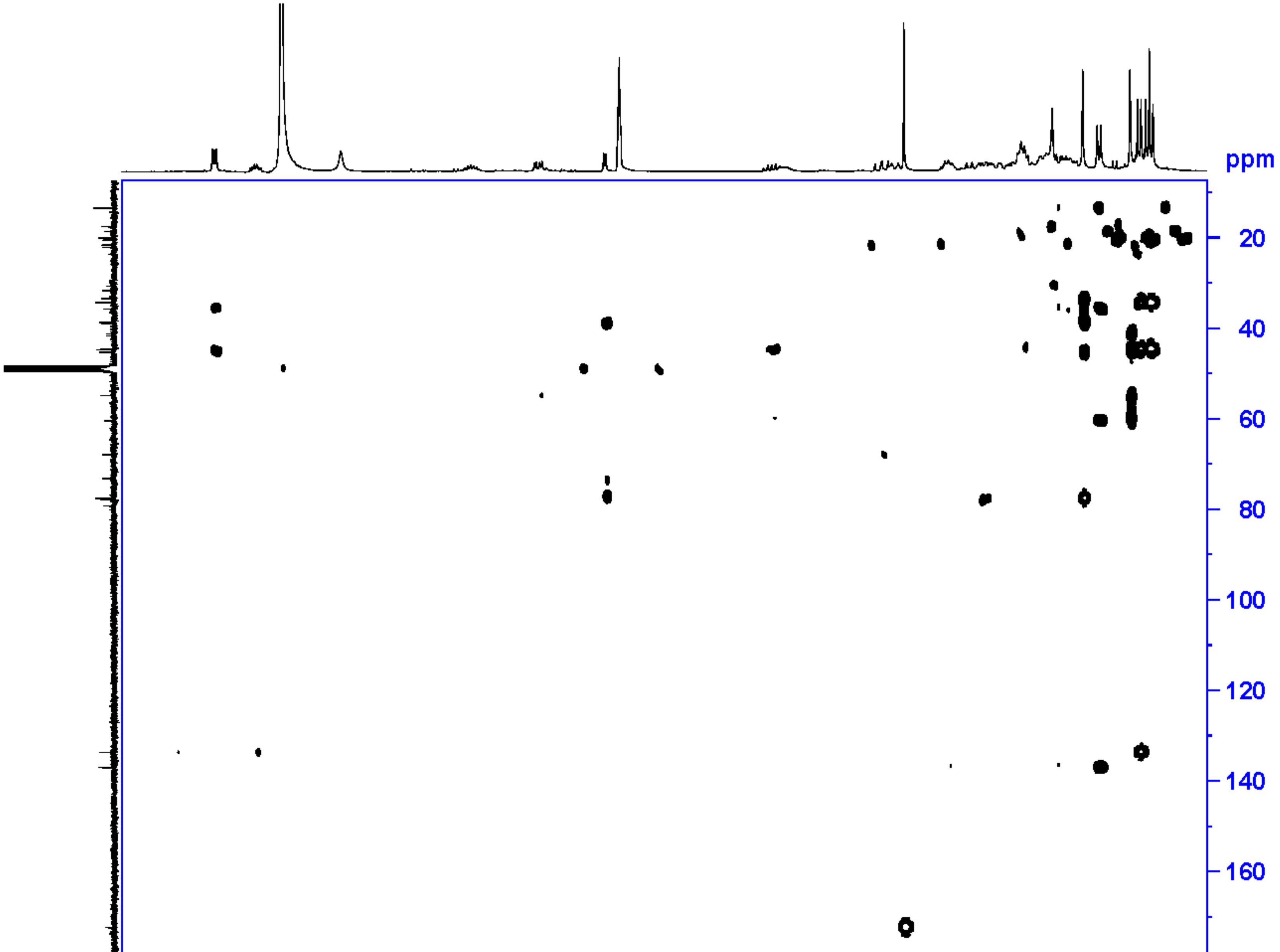


```
Current Data Parameters
NAME           475T4 NEW
EXPNO                  7
PROCNO                 1

F2 - Acquisition Parameters
Date_          20180527
Time               0.09
INSTRUM           spect
PROBHD   5 mm PABBO BB-
PULPROG      hmbcgpndqf
TD                 4096
SOLVENT            MeOD
NS                   48
DS                   16
SWH            4795.396 Hz
FIDRES         1.170751 Hz
AQ            0.4270763 sec
RG                  203
DW              104.267 usec
DE                 6.50 usec
TE                296.1 K
CNST13        8.0000000
D0           0.00000300 sec
D1           1.37220395 sec
D6           0.06250000 sec
D16          0.00020000 sec
IN0          0.00002070 sec

======== CHANNEL f1 ========
NUC1                 1H
P1                13.09 usec
P2                26.18 usec
PL1               -1.00 dB
PL1W        12.14314651 W
SFO1        400.1324008 MHz

======== CHANNEL f2 ========
NUC2                13C
P3                12.37 usec
PL2                1.00 dB
PL2W        28.13319778 W
SFO2        100.6248425 MHz

====== GRADIENT CHANNEL =====
GPNAM[1]       SINE.100
GPNAM[2]       SINE.100
GPNAM[3]       SINE.100
GPZ1              50.00 %
GPZ2              30.00 %
GPZ3              40.10 %
P16             1000.00 usec

F1 - Acquisition parameters
TD                  128
SFO1           100.6248 MHz
FIDRES       188.671585 Hz
SW              240.000 ppm
FnMODE               QF

F2 - Processing parameters
SI                 1024
SF          400.1300068 MHz
WDW                SINE
SSB      0
LB       0 Hz
GB       0
PC                 1.40

F1 - Processing parameters
SI                 1024
[illegible]
```

Figure S24. NOESY spectrum of penicisteroid G (4).

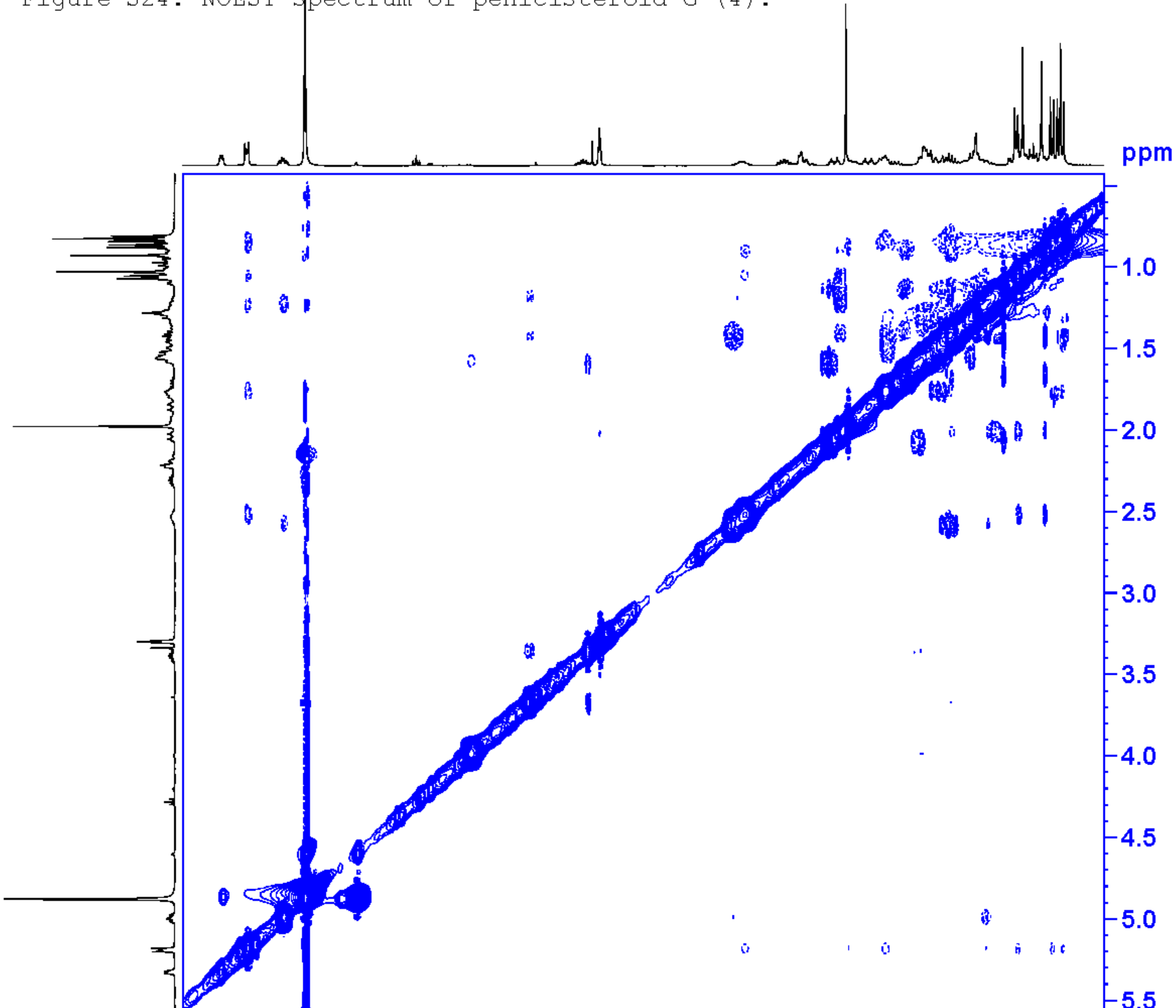


```
Current Data Parameters
NAME              475T4 NEW
EXPNO                     8
PROCNO                    1

F2 - Acquisition Parameters
Date_              20180527
Time                   3.21
INSTRUM               spect
PROBHD   5 mm PABBO BB-
PULPROG            noesyph
TD                     2048
SOLVENT                MeOD
NS                       40
DS                        4
SWH              4795.396 Hz
FIDRES           2.341502 Hz
AQ              0.2135381 sec
RG                      203
DW                  104.267 usec
DE                     6.50 usec
TE                    296.1 K
D0              0.00008747 sec
D1              2.00000000 sec
D8              0.30000001 sec
IN0             0.00020825 sec

======== CHANNEL f1 ========
NUC1                     1H
P1                    13.09 usec
PL1                   -1.00 dB
PL1W            12.14314651 W
SFO1            400.1324008 MHz

F1 - Acquisition parameters
TD                      256
SFO1               400.1324 MHz
FIDRES            18.756207 Hz
SW                   12.000 ppm
FnMODE          States-TPPI

F2 - Processing parameters
SI                     1024
SF              400.1300068 MHz
WDW                   QSINE
SSB                       2
LB         0 Hz
GB         0
PC                     1.00

F1 - Processing parameters
SI                     1024
MC2             States-TPPI
SF              400.1300068 MHz
```

Figure S25. 1H-NMR spectrum of penicisteroid H (5)

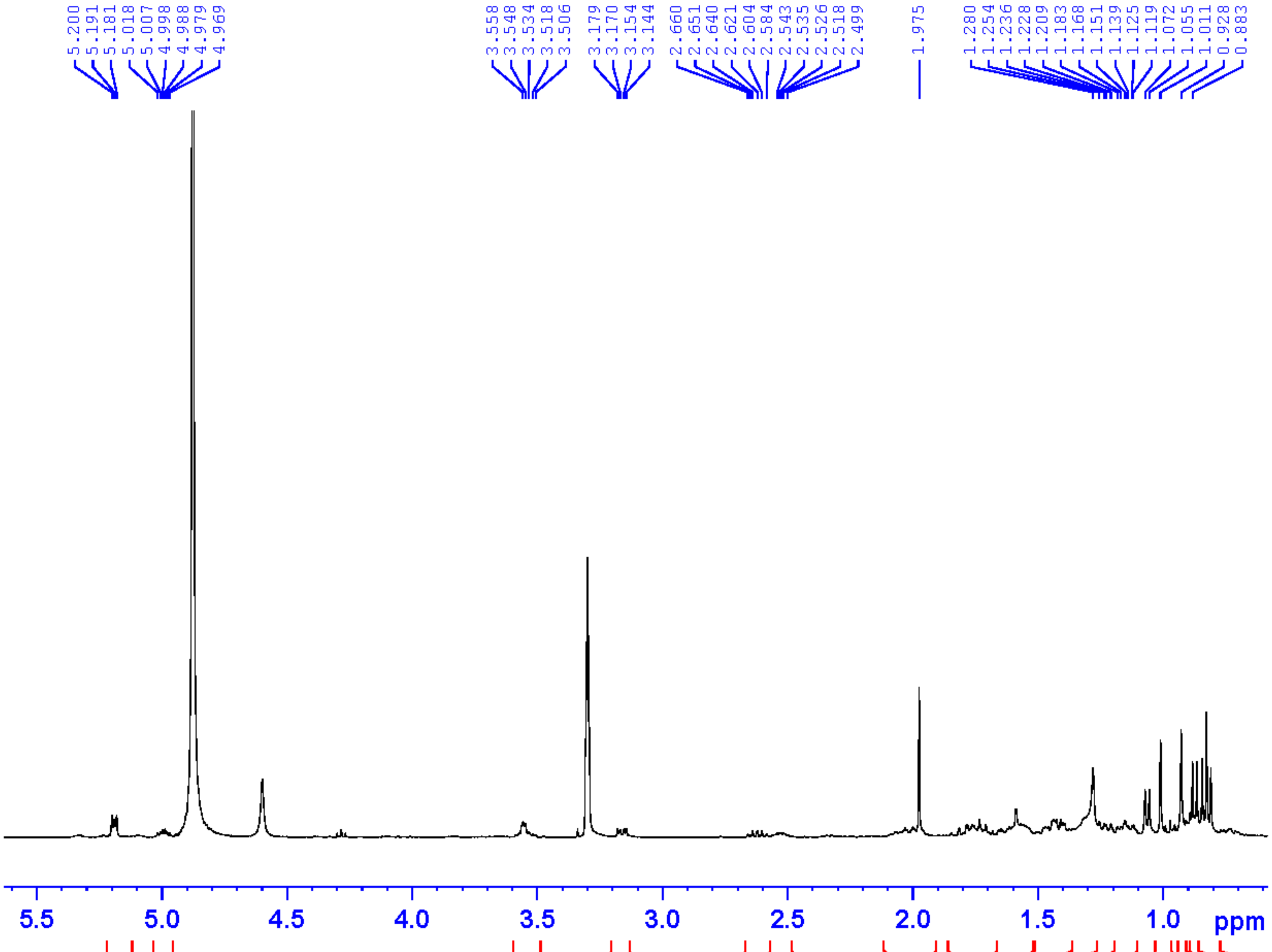

Current Data Parameters
NAME 475T48 D NEW
EXPNO 1
PROCNO 1

F2 - Acquisition Parameters
Date_ 20180509
Time 21.49
INSTRUM spect
PROBHD 5 mm PABBO BB-
PULPROG zg30
TD 65536
SOLVENT MeOD
NS 49
DS 0
SWH 8223.685 Hz
FIDRES 0.125483 Hz
AQ 3.9845889 sec
RG 203
DW 60.800 usec
DE 6.50 usec
TE 296.1 K
D1 2.00000000 sec
TD0 1

======== CHANNEL f1 ========
NUC1 1H
P1 13.09 usec
PL1 -1.00 dB
PL1W 12.14314651 W
SFO1 400.1324710 MHz

F2 - Processing parameters
SI 32768
SF 400.1300065 MHz
WDW EM
SSB 0
LB 0.30 Hz
GB 0
PC 1.00

Figure S26. 13C-NMR spectrum of penicisteroid H (5)

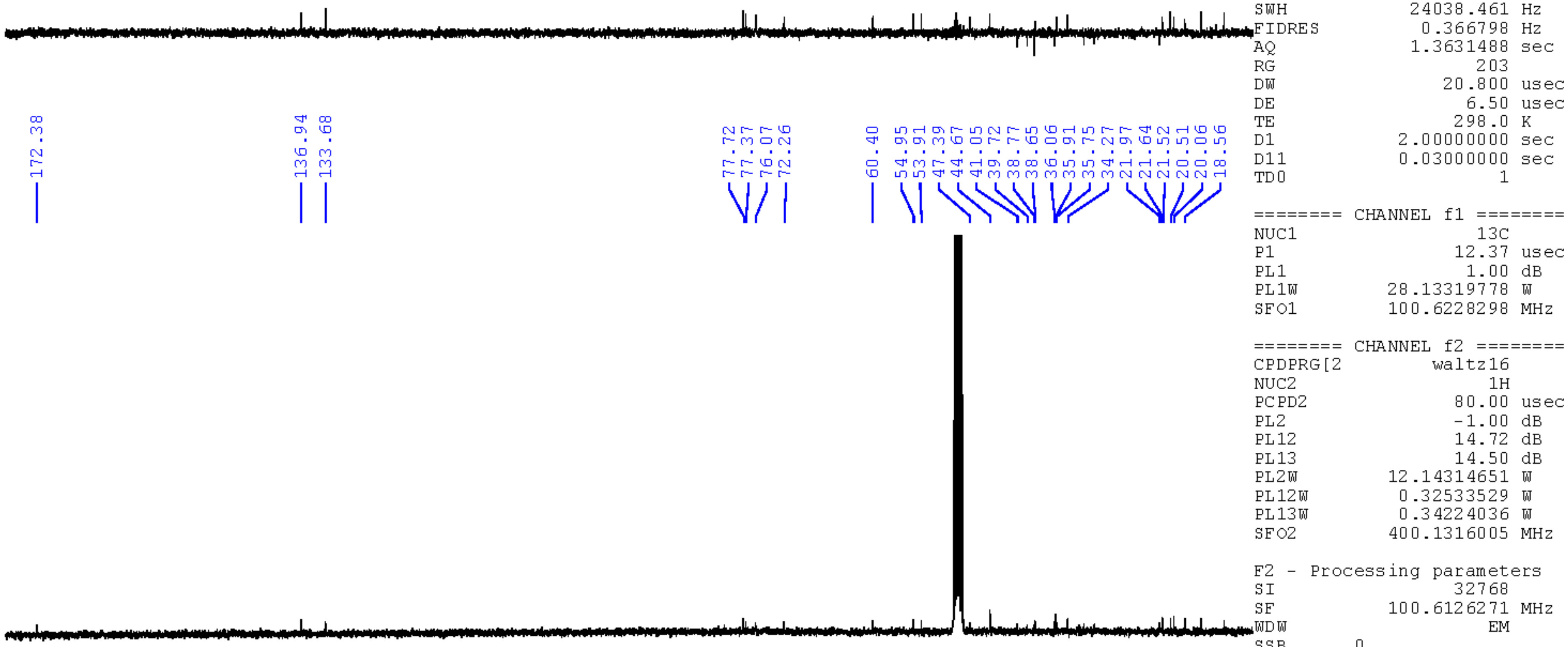


```
Current Data Parameters
NAME        475T48 D NEW
EXPNO                  2
PROCNO                 1

F2 - Acquisition Parameters
Date_           20180531
Time                8.37
INSTRUM            spect
PROBHD    5 mm PABBO BB-
PULPROG          zgpg30
TD                 65536
SOLVENT             MeOD
NS                 12800
DS                     4
SWH            24038.461 Hz
FIDRES          0.366798 Hz
AQ             1.3631488 sec
RG                   203
DW                20.800 usec
DE                  6.50 usec
TE                 298.0 K
D1            2.00000000 sec
D11           0.03000000 sec
TD0                    1

======== CHANNEL f1 ========
NUC1                 13C
P1                 12.37 usec
PL1                 1.00 dB
PL1W         28.13319778 W
SFO1         100.6228298 MHz

======== CHANNEL f2 ========
CPDPRG[2         waltz16
NUC2                  1H
PCPD2              80.00 usec
PL2                -1.00 dB
PL12               14.72 dB
PL13               14.50 dB
PL2W         12.14314651 W
PL12W         0.32533529 W
PL13W         0.34224036 W
SFO2         400.1316005 MHz

F2 - Processing parameters
SI                 32768
SF           100.6126271 MHz
WDW                   EM
SSB        0
```

Figure S27. HSQC spectrum of penicisteroid H (5)

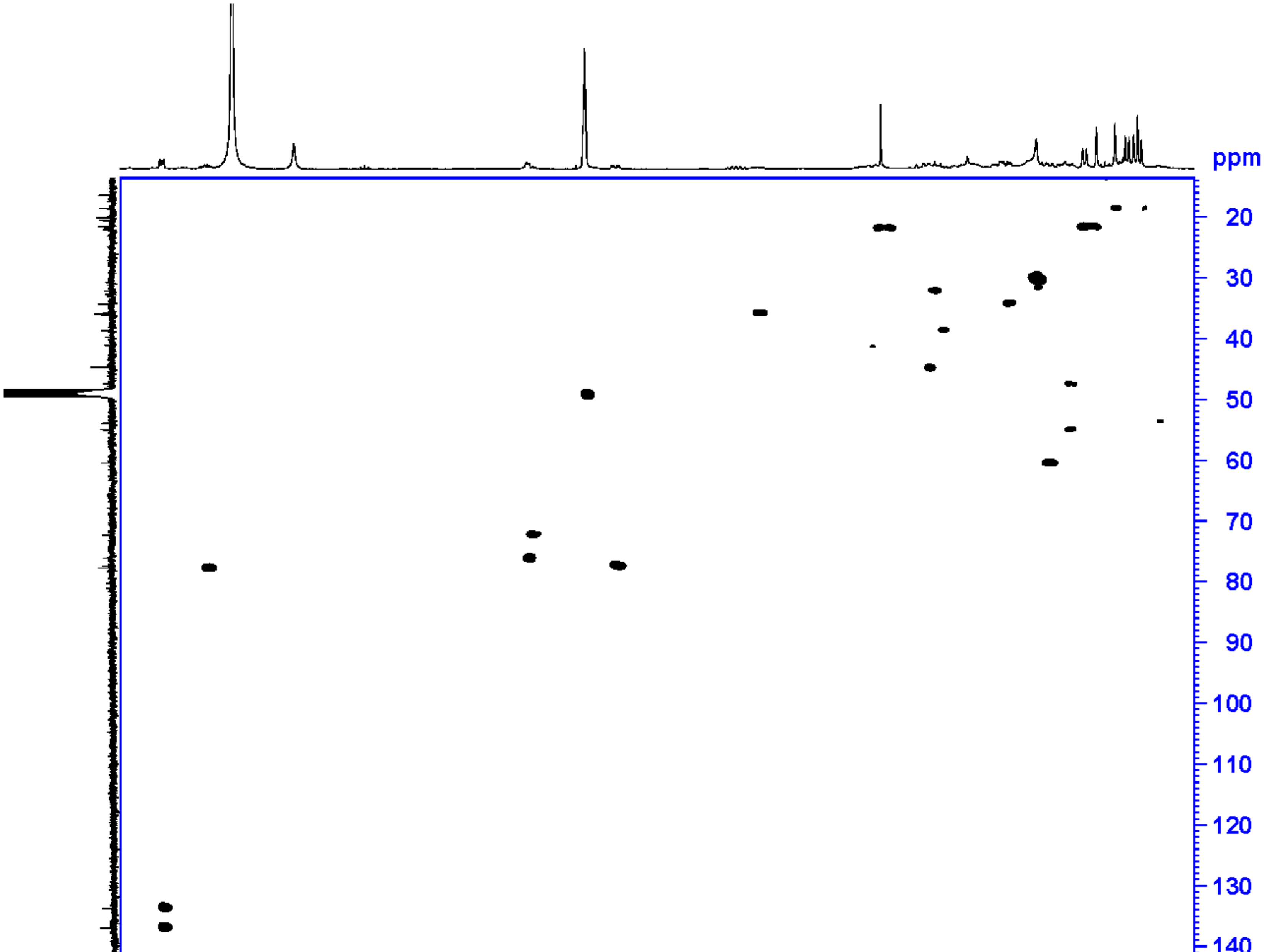


```
Current Data Parameters
NAME        475T48 D NEW
EXPNO                  8
PROCNO                 1

F2 - Acquisition Parameters
Date_           20180616
Time               10.08
INSTRUM            spect
PROBHD   5 mm PABBO BB-
PULPROG        hsqcedetgp
TD                  1024
SOLVENT             MeOD
NS                   180
DS                    32
SWH             5197.505 Hz
FIDRES          5.075689 Hz
AQ             0.0985088 sec
RG                   203
DW                96.200 usec
DE                  6.50 usec
TE                 296.2 K
CNST2        145.0000000
D0            0.00000300 sec
D1            1.50000000 sec
D4            0.00172414 sec
D11           0.03000000 sec
D13           0.00000400 sec
D16           0.00020000 sec
D21           0.00345000 sec
IN0           0.00002260 sec
ZGOPTNS

======== CHANNEL f1 ========
NUC1                  1H
P1                 13.09 usec
P2                 26.18 usec
P28                 1.00 usec
PL1                -1.00 dB
PL1W         12.14314651 W
SFO1         400.1324008 MHz

======== CHANNEL f2 ========
CPDPRG[2            garp
NUC2                 13C
P3                 12.37 usec
P4                 24.74 usec
PCPD2              75.00 usec
PL2                 1.00 dB
PL12               16.65 dB
PL2W         28.13319778 W
PL12W         0.76598305 W
SFO2         100.6238364 MHz

====== GRADIENT CHANNEL =====
GPNAM[1]        SINE.100
GPNAM[2]        SINE.100
GPZ1               80.00 %
GPZ2               20.10 %
P16              1000.00 usec

F1 - Acquisition parameters
TD                   256
SFO1            100.6238 MHz
FIDRES         86.473610 Hz
SW               220.000 ppm
FnMODE    Echo-Antiecho

F2 - Processing parameters
SI                  1024
SF           400.1300068 MHz
WDW                QSINE
SSB                    2
LB       0 Hz
GB       0
PC                  1.40

F1 - Processing parameters
```

Figure S28. COSY spectrum of penicisteroid H (5)

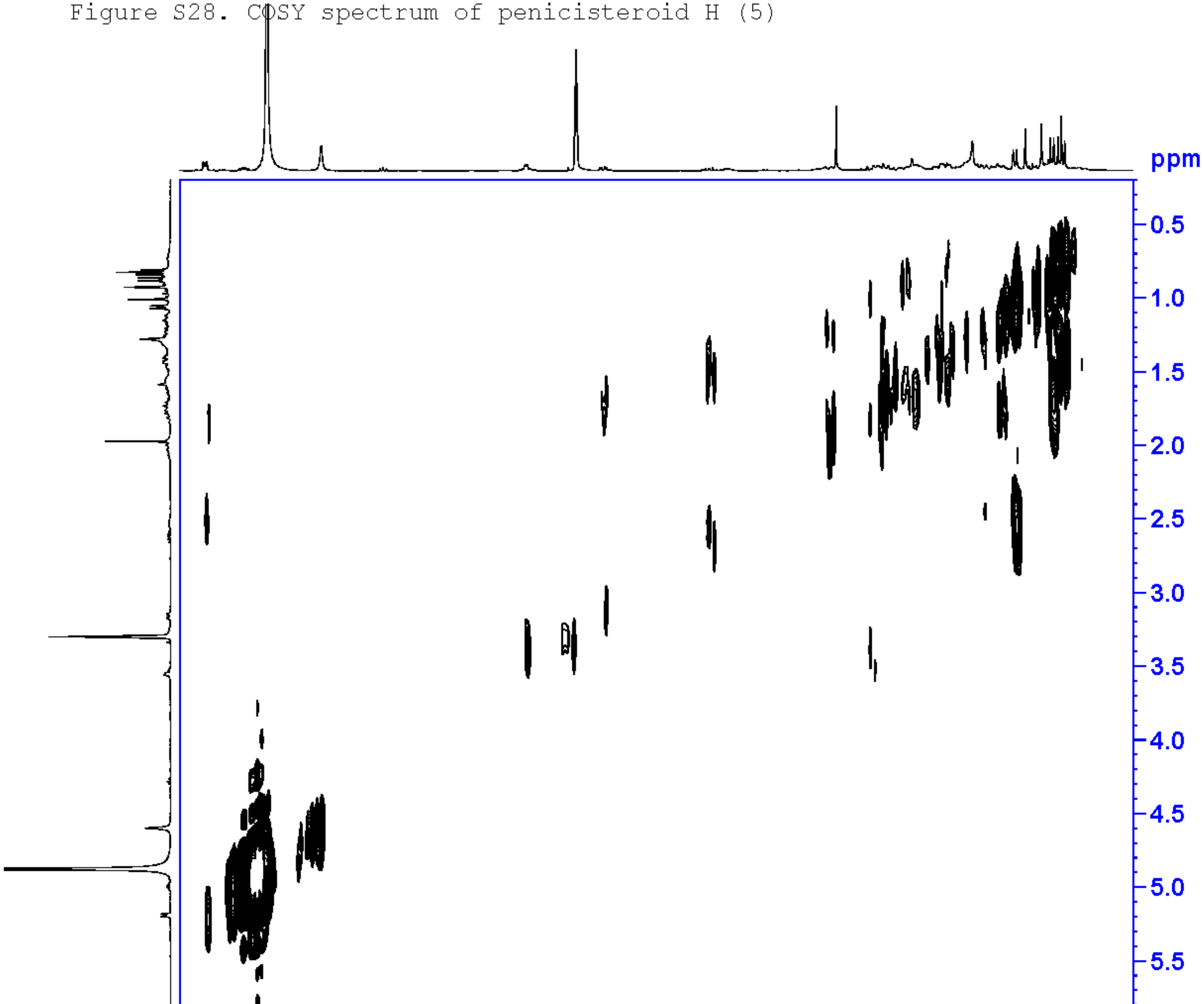


```
Current Data Parameters
NAME        475T48 D NEW
EXPNO                  6
PROCNO                 1

F2 - Acquisition Parameters
Date_           20180614
Time                6.59
INSTRUM            spect
PROBHD   5 mm PABBO BB-
PULPROG       cosygpmfqf
TD                  2048
SOLVENT             MeOD
NS                    32
DS                     8
SWH             4795.396 Hz
FIDRES          2.341502 Hz
AQ             0.2135381 sec
RG                   203
DW               104.267 usec
DE                  6.50 usec
TE                 295.9 K
D0            0.00000300 sec
D1            1.93569195 sec
D13           0.00000400 sec
D16           0.00020000 sec
IN0           0.00020825 sec

======== CHANNEL f1 ========
NUC1                  1H
P1                 13.09 usec
PL1                -1.00 dB
PL1W         12.14314651 W
SFO1         400.1324008 MHz

====== GRADIENT CHANNEL =====
GPNAM[1]        SINE.100
GPNAM[2]        SINE.100
GPNAM[3]        SINE.100
GPZ1               16.00 %
GPZ2               12.00 %
GPZ3               40.00 %
P16              1000.00 usec

F1 - Acquisition parameters
TD                    26
SFO1            400.1324 MHz
FIDRES        184.676498 Hz
SW                12.000 ppm
FnMODE                QF

F2 - Processing parameters
SI                  1024
SF           400.1300068 MHz
WDW                 SINE
SSB      0
LB       0 Hz
GB       0
PC                  1.40

F1 - Processing parameters
SI                  1024
MC2                   QF
[illegible]
```

Figure S29. HMBC spectrum of penicisteroid H (5)

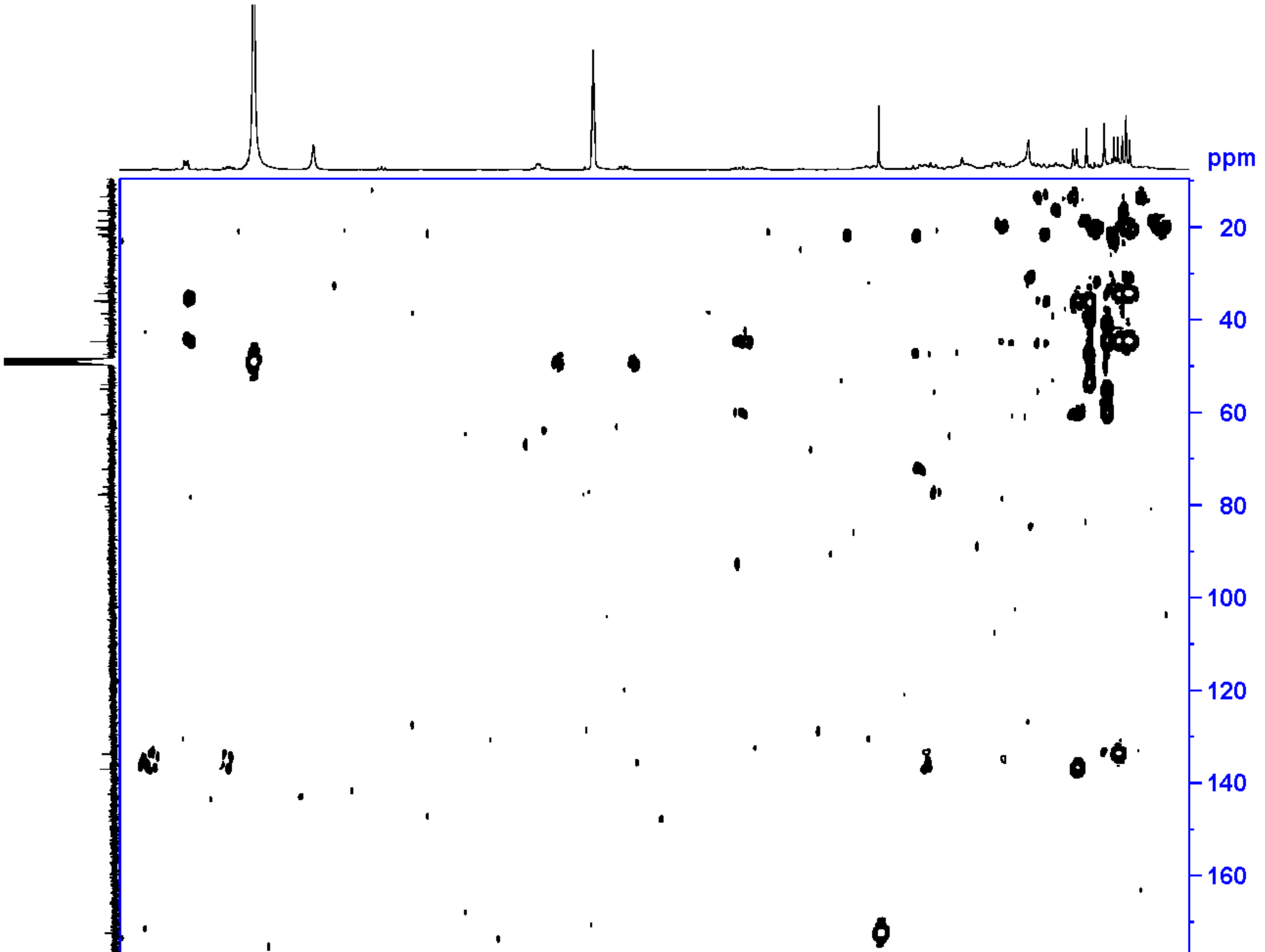

Current Data Parameters
NAME 475T48 D NEW
EXPNO 5
PROCNO 1

F2 - Acquisition Parameters
Date_ 20180613
Time 17.45
INSTRUM spect
PROBHD 5 mm PABBO BB-
PULPROG hmbcgpndqf
TD 4096
SOLVENT MeOD
NS 200
DS 16
SWH 4795.396 Hz
FIDRES 1.170751 Hz
AQ 0.4270763 sec
RG 203
DW 104.267 usec
DE 6.50 usec
TE 296.2 K
CNST13 8.0000000
D0 0.00000300 sec
D1 1.37220395 sec
D6 0.06250000 sec
D16 0.00020000 sec
IN0 0.00002070 sec

======== CHANNEL f1 ========
NUC1 1H
P1 13.09 usec
P2 26.18 usec
PL1 -1.00 dB
PL1W 12.14314651 W
SFO1 400.1324008 MHz

======== CHANNEL f2 ========
NUC2 13C
P3 12.37 usec
PL2 1.00 dB
PL2W 28.13319778 W
SFO2 100.6248425 MHz

====== GRADIENT CHANNEL =====
GPNAM[1] SINE.100
GPNAM[2] SINE.100
GPNAM[3] SINE.100
GPZ1 50.00 %
GPZ2 30.00 %
GPZ3 40.10 %
P16 1000.00 usec

F1 - Acquisition parameters
TD 128
SFO1 100.6248 MHz
FIDRES 188.671585 Hz
SW 240.000 ppm
FnMODE QF

F2 - Processing parameters
SI 1024
SF 400.1300068 MHz
WDW SINE
SSB 0
LB 0 Hz
GB 0
PC 1.40

F1 - Processing parameters
SI 1024
MC2 QF

Figure S30. NOESY spectrum of penicisteroid H (5).

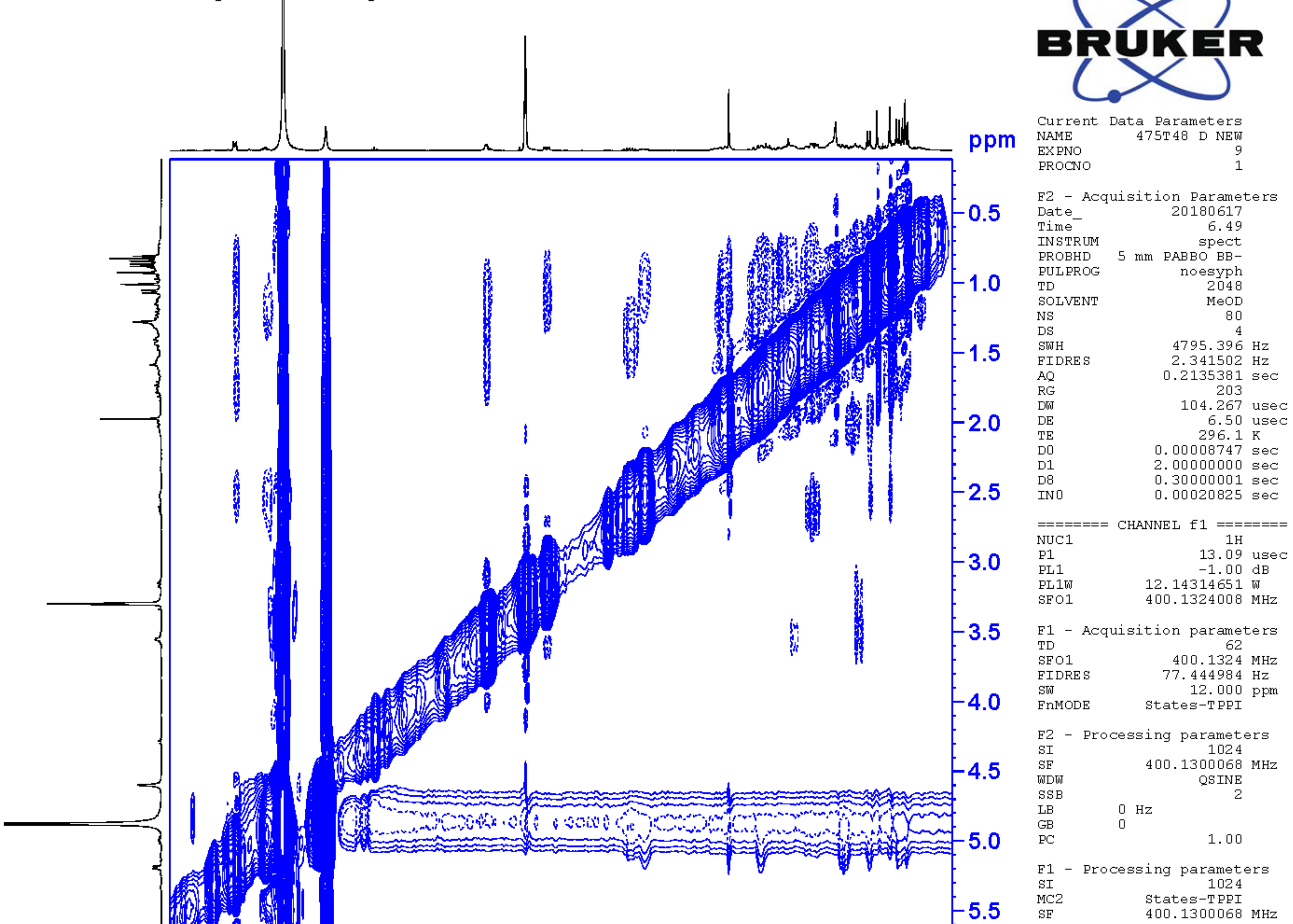

Supplement: Supplementary file 1 [file marinedrugs-17-00178-s001.zip › marinedrugs-461300-SI.pdf]
